# Glucose and insulin levels are associated with arterial stiffness and concentric remodeling of the heart

**DOI:** 10.1186/s12933-019-0948-4

**Published:** 2019-11-04

**Authors:** Marcello Ricardo Paulista Markus, Susanne Rospleszcz, Till Ittermann, Sebastian Edgar Baumeister, Sabine Schipf, Ulrike Siewert-Markus, Roberto Lorbeer, Corinna Storz, Violetta Ptushkina, Annette Peters, Christa Meisinger, Fabian Bamberg, Matthias Nauck, Martin Bahls, Henry Völzke, Stephan Burkhard Felix, Robin Bülow, Wolfgang Rathmann, Marcus Dörr

**Affiliations:** 1 Department of Internal Medicine B, University Medicine Greifswald, Ferdinand-Sauerbruch-Straße, 17475 Greifswald, Germany; 2 German Center for Diabetes Research (DZD), Partner Site Greifswald, Greifswald, Germany; 3 German Centre for Cardiovascular Research (DZHK), Partner Site Greifswald, Greifswald, Germany; 4 Institute of Epidemiology, Helmholtz Zentrum München, German Research Center for Environmental Health, Neuherberg, Germany; 5 Department of Study of Health in Pomerania/Clinical-Epidemiological Research, Institute for Community Medicine, University Medicine Greifswald, Greifswald, Germany; 6 Chair of Epidemiology, Ludwig-Maximilians-Universität München at UNIKA-T Augsburg, Augsburg, Germany; 7 Independent Research Group Clinical Epidemiology, Helmholtz Zentrum München, German Research Center for Environmental Health, Munich, Germany; 8 Institute for Medical Psychology, University Medicine Greifswald, Greifswald, Germany; 9 Department of Radiology, Ludwig-Maximilians-University Hospital, Munich, Germany; 10 Department of Diagnostic and Interventional Radiology, University of Tuebingen, Tübingen, Germany; 11 Institute for Biometrics and Epidemiology, German Diabetes Center (DDZ), Leibniz Center for Diabetes Research at Heinrich Heine University Düsseldorf, Düsseldorf, Germany; 12 German Center for Diabetes Research (DZD), München-Neuherberg, Germany; 13 Department of Diagnostic and Interventional Radiology, Medical Center, University of Freiburg, Faculty of Medicine, University of Freiburg, Freiburg, Germany; 14 Institute of Clinical Chemistry and Laboratory Medicine, University Medicine Greifswald, Greifswald, Germany; 15 Institute of Diagnostic Radiology and Neuroradiology, University Medicine Greifswald, Greifswald, Germany

**Keywords:** Arterial stiffness, Concentric remodeling, Diabetes mellitus, Insulin resistance, Prediabetes

## Abstract

**Background:**

Mortality attributable to heart failure remains high. The prevalence of heart failure in patients with diabetes mellitus ranges from 19 to 26%. It is estimated that up to 21.1 million adults in the United States have diagnosed diabetes mellitus and around 80.8 million have impaired fasting glucose. We investigated the associations of fasting glucose (FG) and fasting insulin (FI), the homeostasis model assessment-insulin resistance index (HOMA-IR) and 2-h postload glucose (2HG) and insulin (2HI) with parameters of left ventricular geometry and function and arterial stiffness determined by magnetic resonance imaging in individuals without diagnosed type 2 diabetes.

**Methods:**

Cross-sectional analyses of 1001 individuals (453 women, 45.3%), aged 21 to 80 years, from two independent population-based studies, the Study of Health in Pomerania (SHIP-TREND-0) and KORA FF4 Study. FG, FI, HOMA-IR, 2HG and 2HI, as well as glucose tolerance categories, were analyzed for associations with heart and arterial parameters using multivariable-adjusted linear regression models.

**Results:**

In total, 390 individuals (39%) had prediabetes (isolated impaired fasting glucose, isolated glucose tolerance or both), and 49 (4.9%) were found to have unknown type 2 diabetes. In the multivariable-adjusted analysis, positive linear associations of FG, FI, HOMA-IR, 2HG and 2HI with arterial stiffness index and left ventricular wall-thickness and concentricity and inverse linear associations with left ventricular end-diastolic volume were observed. A 1 mmol/l higher FG was associated with a 1.18 ml/m^2.7^ (1.80 to 0.57; p < 0.001) lower left ventricular end-diastolic volume index, a 0.042 mm/m^2.7^ (0.014 to 0.070) higher left ventricular wall-thickness index, a 0.12 mmHg m^2.7^/ml (0.06 to 0.17; p < 0.001) greater arterial stiffness index and a 0.037 g/ml (0.018 to 0.056; p < 0.001) higher left ventricular concentricity.

**Conclusions:**

Our findings suggest that higher glucose levels in the prediabetic range and insulin resistance might lead to higher arterial stiffness and concentric remodeling of the heart.

## Introduction

The leading cause of death in patients with type 2 diabetes is cardiovascular disease. Moreover, the risk of cardiovascular mortality is doubled when compared with individuals without type 2 diabetes [1]. One of the major cardiovascular complications of type 2 diabetes is heart failure with a prevalence that ranges from 19 to 26% among patients with diabetes mellitus [2]. In line with these observations, the term diabetic cardiomyopathy was defined as a left ventricular dysfunction that occurs in diabetic patients in the absence of coronary atherosclerosis and hypertension [2]. The initial stage of the diabetic cardiomyopathy is characterized by subclinical changes of the cardiac geometry and marginal changes in diastolic function. A previous analysis [3] of our group showed that 43.1% of adults in the northeast and 30.1% in the south of Germany already presented glucose levels that fulfill the criteria of prediabetes. Importantly, previous studies [4–7] already showed subclinical alterations in cardiac structure and function not just in patients with type 2 diabetes, but already in individuals with prediabetes. Otherwise, the results of these studies are sometimes contradictory regarding their findings. While a previous study of the Multiethnic Study of Atherosclerosis (MESA) [4] showed that individuals with impaired fasting glucose (IFG) had no significant difference regarding left ventricular mass (LVM), when compared with individuals with normal fasting glucose, a more recent analysis of the same cohort [5] demonstrated that subjects with IFG had a higher LVM. Contrary to that, an analysis from the Framingham Heart Study [6], showed that the homeostasis model assessment-insulin resistance index (HOMA-IR) was inversely related with the LVM.

The aim of the present study was to investigate the associations of parameters from an oral glucose tolerance test (OGTT), as well as the presence of prediabetes and unknown type 2 diabetes (UT2D), with indicators of the left ventricular (LV) geometry and function and arterial stiffness as determined by magnetic resonance imaging (MRI) using data from two population-based samples from the Northeastern and Southern part of Germany.

## Materials and methods

### Pooled study sample

The present cross-sectional study is based on data from two independent population-based investigations, the Study of Health in Pomerania (SHIP-TREND-0) [8, 9] and the Cooperative Health Research in the Region of Augsburg (KORA FF4) [10]. Our pooled sample, from SHIP-TREND-0 and KORA FF4, comprised 1391 individuals (604 women, 43.4%) aged 21 to 81 years. Individuals with inadequate MRI image quality (n = 79), previous myocardial infarction or stroke (n = 16), left ventricular ejection fraction (determined by MRI) less than 40% (n = 9), fasting time less than 8 h (n = 217), use of hypoglycemic medication (n = 37), missing values for OGTT parameters (n = 17) or any of the covariates (n = 9) as well as individuals with extreme values (> 99.5th percentile for fasting glucose, insulin or 2-h postload glucose; n = 6) were excluded. Accordingly, our final analytical sample consisted of 1001 individuals (453 women, 45.3%), aged 21 to 80 years.

All study participants gave written informed consent. The study was approved by the ethics committees of the University of Greifswald, the Bavarian Chamber of Physicians, and the Ludwig-Maximilians-Universität München and complies with the Declaration of Helsinki.

### Glucose and insulin measurements, oral glucose tolerance test and classification of prediabetes and unknown type 2 diabetes

Measurements of fasting glucose (FG) and 2-h postload glucose (2HG) were based on plasma in SHIP-TREND-0 and on serum in KORA FF4. Duplicate measurements were carried out using serum samples from all SHIP-TREND-0 participants and serum glucose from KORA FF4 and plasma glucose from SHIP-TREND-0 were considered as comparable for the current analysis (concordance correlation coefficient of r = 0.94 in a validation study comparing plasma and serum glucose measurements).

In both studies, FG was sampled and 75 g of anhydrous glucose (Dextro OGT; Boehringer Mannheim, Mannheim, Germany) was given to those participants without diagnosed type 2 diabetes or taking glucose-lowering agents. In SHIP-TREND-0, plasma FG and 2HG levels were measured using a hexokinase method (Dimension Vista 1500, Siemens Healthcare Diagnostics, Eschborn, Germany) [3] and serum fasting insulin (FI) and 2-h postload glucose insulin (2HI) values were assessed by an electrochemiluminescence immunoassay (ADVIA Centaur, Siemens Healthcare Diagnostics, Eschborn, Germany) [11]. In KORA FF4, serum FG and 2HG levels were measured using an enzymatic colorimetric method (Dimension Vista 1500, Siemens Healthcare Diagnostics, Eschborn, Germany or Cobas c702, Roche Diagnostics GmbH, Mannheim, Germany) and FI and 2HI values were measured by a solid-phase enzyme-labeled chemiluminescent immunometric assay (Immulite 2000 Xpi, Siemens Healthcare Diagnostics, Eschborn, Germany) or by an electrochemiluminescence immunoassay (Cobas e 602, Roche Diagnostics GmbH, Mannheim, Germany).

The homeostasis model assessment-insulin resistance index (HOMA-IR) was calculated as (FG [mmol/l] × FI [μU/ml])/22.5 [12].

Following the criteria of the American Diabetes Association (ADA) [13], we classified individuals as having normal glucose tolerance (NGT) when they had FG values < 5.6 mmol/l (< 100 mg/dl) and 2HG < 7.8 mmol/l (< 140 mg/dl). Unknown type 2 diabetes (UT2D) was defined as FG values ≥ 7.0 mmol/l (≥ 126 mg/dl) or 2HG ≥ 11.1 mmol/l (≥ 200 mg/dl). Participants were classified as having prediabetes if FG values were between 5.6 and 6.9 mmol/l (100–125 mg/dl, impaired fasting glucose: IFG) and/or 2HG values were between 7.8 and 11.0 mmol/l (140–199 mg/dl, impaired glucose tolerance: IGT) [3, 13]. We defined three groups of prediabetes: isolated impaired fasting glucose (i-IFG), isolated impaired glucose tolerance (i-IGT), and combined IFG and IGT (IFG + IGT) [3, 13].

### Cardiac MR imaging

In SHIP-TREND-0, cardiac MR imaging was performed on a 1.5 Tesla MR system (Magnetom Avanto; Siemens Medical Systems, Erlangen, Germany) [11, 14] and in KORA FF4, on a 3 Tesla MR system (Magnetom Skyra; Siemens Medical Systems, Erlangen, Germany) [15, 16]. In both studies imaging of cardiac function and morphology was performed using cine steady-state free precession (cine-SSFP) sequences.

### Image analysis

LV end-diastolic volume (LVEDV) was determined during the first image of the acquisition. LV end-systolic volume (LVESV) was measured by determining the phase in which the LV intra-cavity blood pool was at its smallest by visual assessment at the midventricular level. LV myocardial mass (LVM) was calculated at the end-diastole using the specific density of the myocardium (1.05 g/cm^3^) [14]. Papillary muscles were included in the LVM and excluded of the LV end-diastolic and systolic volumes. Basal slices were included if at least half of the LV circumference blood pool was confined by myocardium [17]. Inclusion or exclusion of apical slices depended on the visibility of myocardium. LV wall-thickness (LVWT) was determined in the 16-segment model (according to the AHA-segment model) [18]. LV concentricity (LVC) was calculated as LVM/LVEDV. LV stroke volume (LVSV), LV cardiac output (LVCO) and LV ejection fraction (LVEF) were calculated following the formulas below:

$${\text{LVSV }}\left( {\text{ml}} \right) \, = {\text{ LVEDV }}{-}{\text{ LVESV}}$$

$${\text{LVCO }}\left( {{\text{l}}/{ \hbox{min} }} \right) \, = {\text{ LVSV }} \times {\text{ heart rate}}$$

$${\text{LVEF }}\left( \% \right) \, = \, \left( {{\text{LVEDV }} - {\text{ LVESV}}} \right) \, /{\text{ LVEDV}}$$

LVM, LVEDV, LVESV, LVWT, LVSV and LVCO were indexed for body height in meters, normalized to the allometric power of 2.7, which linearizes the relations between the cardiac anatomic and functional parameters with height and identifies the impact of obesity [19]. This resulted in LVM index (LVMI), LVEDV index (LVEDVI), LVESV index (LVESVI), LVWT index (LVWTI), LVSV index (LVSI) and LVCO index (LVCI).

Arterial stiffness index (ASI) was calculated as (systolic blood pressure − diastolic blood pressure)/LVSI [20].

### Interview, medical and laboratory examinations

In both studies, information on socio-economic variables (including years of school education [< 10, 10, or > 10 years]), smoking status (never, former or current smoker) [21], alcohol consumption (in grams per day) and medical history was collected by trained and certificated medical staff during a standardized interview. Sedentary lifestyle was defined as individuals who did not participate in leisure time exercise, for at least 1 h/week, during summer or winter [22]. Participants were asked to bring the original packaging of their medications that were taken during the last 7 days before the examination date. Unique identifiers and drug names were recorded according to the ATC classification system.

All participants underwent an extensive standardized medical examination. Anthropometric measurements included height and weight based on recommendations of the World Health Organization (WHO) [23]. Weight was measured to the nearest 0.1 kg in light clothing and without shoes using standard digital scales. Body mass index (BMI) was calculated as weight (kg)/height^2^ (m^2^). Waist circumference (WC) was measured to the nearest 0.1 cm using an inelastic tape midway between the lower rib margin and the iliac crest in the horizontal plane, with the participant standing comfortably with weight distributed evenly on both feet [24]. While in SHIP-TREND-0 body fat-free mass (FFM) and fat mass (FM) were measured by bioelectrical impedance analysis (BIA) using a multifrequency Nutriguard-M device (Data Input, Pöcking, Germany) and the NUTRI4 software (Data Input, Pöcking, Germany) [25–27], in KORA FF4, BIA scans were obtained by BIA 2000-S device (Data Input, Pöcking, Germany) with an operating frequency of 50 kHz at 0.8 mA. Ohmic resistance was measured at the dominant hand (between wrist and dorsum) and the dominant foot (between angle and dorsum).

After a resting period of at least 5 min, systolic and diastolic blood pressures as well as heart rate were measured three times on the right arm of seated subjects using an oscillometric digital blood pressure monitor (HEM-705CP, Omron Corporation, Tokyo, Japan) with an interval of 3 min between readings. The mean of the second and third measurements was used for the present analyses. Antihypertensive medication was defined as use of agents with the ATC-code C02, C03, C07, C08 and C09 [28]. Hypertension was defined as systolic blood pressure ≥ 140 mmHg and/or diastolic blood pressure ≥ 90 mmHg and/or current self-reported use of any anti-hypertensive medications.

Fasting blood samples were obtained from all study participants while sitting [29]. In SHIP-TREND-0, glycated hemoglobin was determined by high-performance liquid chromatography (Diamat, Bio-Rad Laboratories, Munich, Germany). Total serum cholesterol, low-density lipoprotein cholesterol (LDL-C) and high-density lipoprotein cholesterol (HDL-C) were measured photometrically (Dimension RxL or Dimension VISTA 1500, Siemens Healthcare Diagnostics, Eschborn, Germany). Serum creatinine concentration was assessed using a modified kinetic Jaffé method (Dimension RxL or Dimension Vista 1500, Siemens Healthcare Diagnostics, Eschborn, Germany). In KORA FF4, glycated hemoglobin was measured in hemolyzed whole blood using the cation-exchange high performance liquid chromatographic, photometric VARIANT II TURBO HbA1c Kit-2.0 assay on a VARIANT II TURBO Hemoglobin Testing System (Bio-Rad Laboratories Inc., Hercules, USA). Total serum cholesterol, low-density lipoprotein cholesterol (LDL-C), high-density lipoprotein cholesterol (HDL-C) and serum creatinine concentrations were measured using an enzymatic colorimetric method (Dimension Vista 1500, Siemens Healthcare Diagnostics, Eschborn, Germany or Cobas c702, Roche Diagnostics GmbH, Mannheim, Germany). Because of the changes from Siemens to Roche, the Siemens measurement results were calibrated to the Roche measurements using the following formulas (in mg/dl): Total_Cholesterol_Roche = 3.00 + (Total_Cholesterol_Siemens * 1.00); HDL_Cholesterol_Roche = 2.40 + (HDL_Cholesterol_Siemens * 1.12); LDL_Cholesterol_Roche = antilog (− 0.13328 + [log LDL_Cholesterol_Siemens * 1.03051]); Creatine_Roche = − 0.037568 + (Creatinine_Siemens * 1.02703) [16].

Hypercholesterolemia was defined as use of lipid-lowering medication defined by the ATC-code C10 and/or total serum cholesterol ≥ 6.2 mmol/l and/or LDL-C ≥ 4.1 mmol/l and/or total cholesterol/HDL-C ratio ≥ 5.0. The estimated glomerular filtration rate was estimated according to the CKD-EPI formula [30] and expressed in ml/min/1.73 m^2^.

### Statistical analysis

To characterize the study population, data was reported as median (with 25th and 75th percentiles) for continuous variables and as percentages for categorical variables stratified by OGTT classification.

We used linear regression models to associate FG, FI, HOMA-IR, 2HG and 2HI levels and OGTT groups with LVMI, LVEDVI, LVESVI, LVWTI, LVC, ASI, LVSI, HR, LVCI and LVEF. The basic multivariable models were adjusted for age, sex, body fat-free mass and body fat mass (both assessed by BIA), systolic blood pressure, use of antihypertensive medication, smoking status, alcohol consumption, sedentarism (defined as individuals who did not participate in leisure time exercise for at least 1 h/week during summer or winter [22]), estimated glomerular filtration rate, fasting time and study sample (SHIP-TREND-0, KORA FF4). We used fractional polynomials to test potential non-linear relationships between exposure and outcomes [31].

A two-sided p-value p < 0.05 was considered as statistically significant. Statistical analyses were performed using Stata 14.2 (Stata Corporation, College Station, TX, USA).

Please see Additional file 1 for a more detailed description.

## Results

Among the total study sample of 1001 individuals (453 women, 45.3%), aged 21 to 80 years, 39.0% of the subjects had prediabetes (isolated IFG, isolated IGT and combined IFG and IGT). Out of all prediabetes subjects, 60.8% had isolated IFG, 23.1% had combined IFG and IGT and 16.1% had isolated IGT. In addition, the percentage of individuals with UT2D was 4.9%.

Table 1 shows the clinical characteristics of the study sample stratified by OGTT classification. Individuals with NGT were younger, more likely female, had a lower body mass index (BMI), body fat-free mass, fat mass and waist circumference and were less likely to have a history of hypertension and hypercholesterolemia, with a concomitant less frequent use of antihypertensive and lipid-lowering medication. They also had higher eGFR and were more often current smokers.

**Table 1:** Characteristics of the study sample stratified by oral glucose tolerance test (OGTT) classification: normal glucose tolerance (NGT), isolated impaired fasting glucose (i-IFG), isolated impaired glucose tolerance (i-IGT), combined IFG and IGT (IFG + IGT) and unknown type 2 diabetes (UT2D)

Parameter	NGT	i-IFG	i-IGT	IFG + IGT	UT2D	p-value*
N (%)	562 (56.1)	237 (23.7)	63 (6.29)	90 (8.99)	49 (4.90)	
Age (years)	47 (39, 57)	55 (46, 62)	51 (41, 62)	60 (52, 66)	61 (54, 68)	*<* *0.001*
Women (%)	52.7	32.5	55.6	31.1	34.7	*<* *0.001*
Fasting serum glucose (mmol/l)	5.1 (4.9, 5.3)	5.9 (5.7, 6.2)	5.2 (4.9, 5.4)	6.0 (5.7, 6.3)	7.2 (6.3, 7.5)	*<* *0.001*
2-h postload serum glucose (mmol/l)	5.3 (4.6, 6.2)	6.0 (5.4, 6.8)	8.4 (8.1, 9.1)	8.8 (8.2, 9.5)	11.6 (10.1, 13.9)	*<* *0.001*
Fasting insulin (µlU/ml)	7.3 (5.2, 9.8)	10.7 (7.3, 14.9)	12.3 (7.1, 15.6)	14.4 (10.8, 19.0)	18.1 (13.5, 26.2)	*<* *0.001*
2-h postload insulin (µlU/ml)	37.0 (24.0, 57.0)	50.0 (32.0, 73.9)	97.6 (68.0, 160)	131 (75.5, 167)	132 (91.0, 187)	*<* *0.001*
Homeostasis model assessment-insulin resistance index (HOMA-IR)	1.65 (1.18, 2.25)	2.76 (1.91, 4.03)	2.69 (1.68, 3.63)	3.76 (2.86, 5.19)	5.71 (3.51, 7.94)	*<* *0.001*
Glycated hemoglobin (%)	5.2 (4.9, 5.4)	5.4 (5.2, 5.6)	5.3 (4.9, 5.6)	5.6 (5.2, 5.9)	6.0 (5.5, 6.4)	*<* *0.001*
Estimated glomerular filtration rate (ml/min/1.73 m^2^)	93.9 (84.0, 103)	89.5 (79.8, 97.4)	87.7 (79.0, 100)	84.9 (73.6, 96.3)	88.1 (79.9, 96.1)	*<* *0.001*
Smoking (%)						
Never	40.8	38.4	41.3	40.0	49.0	
Current	23.5	18.6	11.1	14.4	14.3	
Former	35.8	43.0	47.6	45.6	36.7	0.080
Alcohol consumption (g/day)	4.25 (1.06, 13.4)	8.79 (2.69, 22.9)	2.90 (0.34, 12.9)	6.33 (1.45, 17.8)	4.69 (1.14, 12.9)	*0.003*
Weight (kg)	76.2 (65.8, 85.4)	84.4 (75.8, 94.0)	81.9 (74.3, 95.0)	89.3 (77.5, 96.1)	90.9 (81.2, 98.5)	*<* *0.001*
Height (cm)	171 (164, 179)	174 (165, 180)	170 (164, 175)	172 (167, 178)	173 (165, 176)	0.064
Body mass index (kg/m^2^)	25.5 (23.3, 28.4)	28.1 (25.8, 30.7)	28.9 (26.6, 32.1)	29.3 (27.1, 31.7)	31.1 (27.7, 33.1)	*<* *0.001*
Body fat-free mass (kg)	52.5 (45.4, 64.2)	61.1 (51.6, 68.1)	55.7 (50.0, 60.8)	63.7 (53.2, 68.3)	61.3 (51.9, 68.1)	*<* *0.001*
Body fat mass (kg)	20.5 (16.5, 26.1)	23.8 (19.5, 29.2)	28.8 (20.3, 33.9)	25.6 (20.9, 32.2)	29.0 (23.2, 34.3)	*<* *0.001*
Waist circumference (cm)	86.2 (78.0, 95.2)	96.0 (88.1, 104)	94.5 (89.5, 106)	101 (94.0, 109)	104 (93.2, 112)	*<* *0.001*
Systolic blood pressure (mmHg)	120 (109, 130)	126 (116, 137)	128 (113, 139)	133 (123, 144)	137 (126, 150)	*<* *0.001*
Diastolic blood pressure (mmHg)	74.0 (68.0, 80.0)	77.5 (72.5, 83.5)	78.5 (72.5, 86.0)	81.5 (75.0, 89.0)	80.5 (72.5, 86.5)	*<* *0.001*
Hypertension (%)	23.1	45.2	52.4	67.8	77.6	*<* *0.001*
Antihypertensive medications (%)	14.1	33.3	25.4	44.4	55.1	*<* *0.001*
Total cholesterol (mmol/l)	5.40 (4.80, 6.07)	5.60 (5.00, 6.38)	5.70 (4.90, 6.07)	4.16 (3.58, 4.80)	5.70 (4.90, 6.50)	*<* *0.001*
Hypercholesterolemia (%)	32.7	51.9	44.4	54.4	57.1	*<* *0.001*
Lipid-lowering medication (%)	4.27	10.6	14.3	3.33	18.4	*<* *0.001*
Sedentarism (%)	30.4	32.5	33.3	36.7	38.8	0.619

Data are medians (25th, 75th percentile) or percentage

Italic values indicate significance of p-value < 0.05

* p-values are based on the Chi-squared test for categorical variables and the Wilcoxon rank-sum (or Mann–Whitney) tests for continuous variables

### Associations of FG, FI, HOMA-IR, 2HG and 2HI and the OGTT groups with LV geometry

**Fig. 1:**
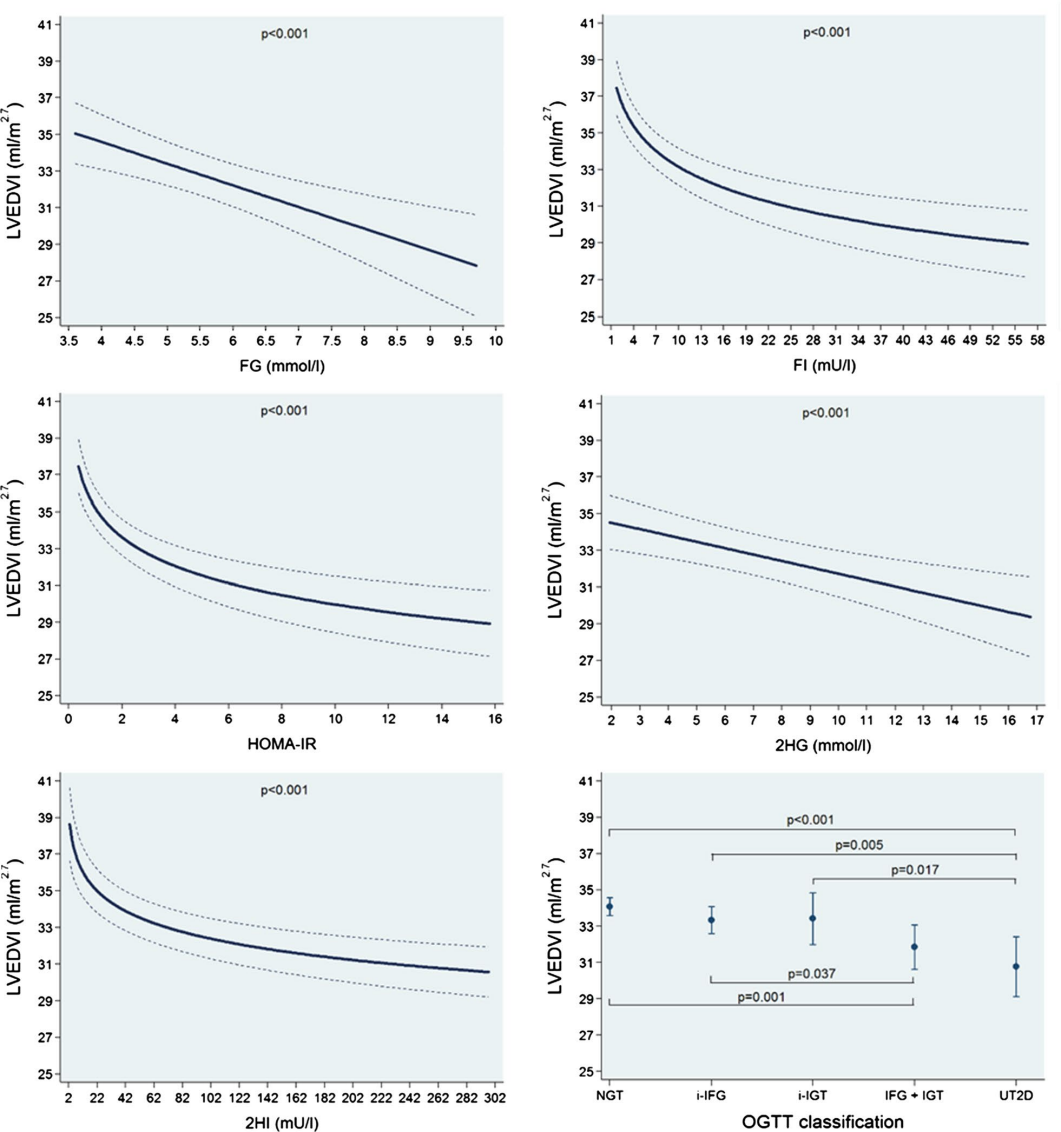
Adjusted* line (95% CI) showing the associations between fasting glucose (FG) and insulin (FI), the homeostasis model assessment-insulin resistance index (HOMA-IR) and 2-h postload glucose (2HG) and insulin (2HI) with left ventricular end-diastolic volume index (LVEDVI). Adjusted* mean (95% CI) LVEDVI according to oral glucose tolerance test (OGTT) classification: normal glucose tolerance (NGT), isolated impaired fasting glucose (i-IFG), isolated impaired glucose tolerance (i-IGT), combined IFG and IGT (IFG + IGT) and unknown type 2 diabetes (UT2D). *Linear regression adjusted for age, sex, body fat-free mass, body fat mass, systolic blood pressure, use of antihypertensive medication, smoking status, alcohol consumption, sedentarism, estimated glomerular filtration rate, fasting time and study sample

**Fig. 2:**
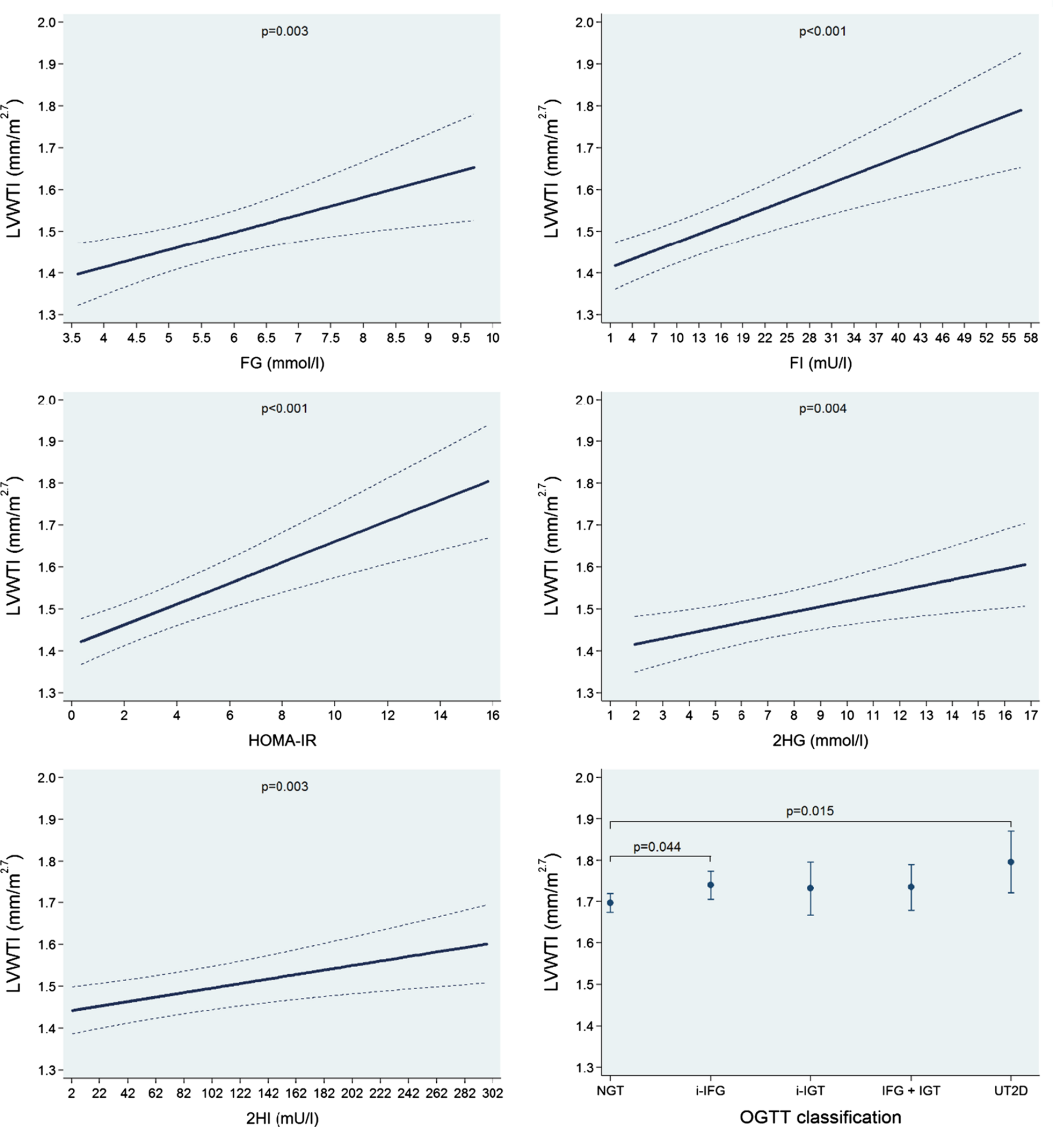
Adjusted* line (95% CI) showing the associations between fasting glucose (FG) and insulin (FI), the homeostasis model assessment-insulin resistance index (HOMA-IR) and 2-h postload glucose (2HG) and insulin (2HI) with left ventricular wall-thickness index (LVWTI). Adjusted* mean (95% CI) LVWTI according to oral glucose tolerance test (OGTT) classification: normal glucose tolerance (NGT), isolated impaired fasting glucose (i-IFG), isolated impaired glucose tolerance (i-IGT), combined IFG and IGT (IFG + IGT) and unknown type 2 diabetes (UT2D). *Linear regression adjusted for age, sex, body fat-free mass, body fat mass, systolic blood pressure, use of antihypertensive medication, smoking status, alcohol consumption, sedentarism, estimated glomerular filtration rate, fasting time and study sample

**Fig. 3:**
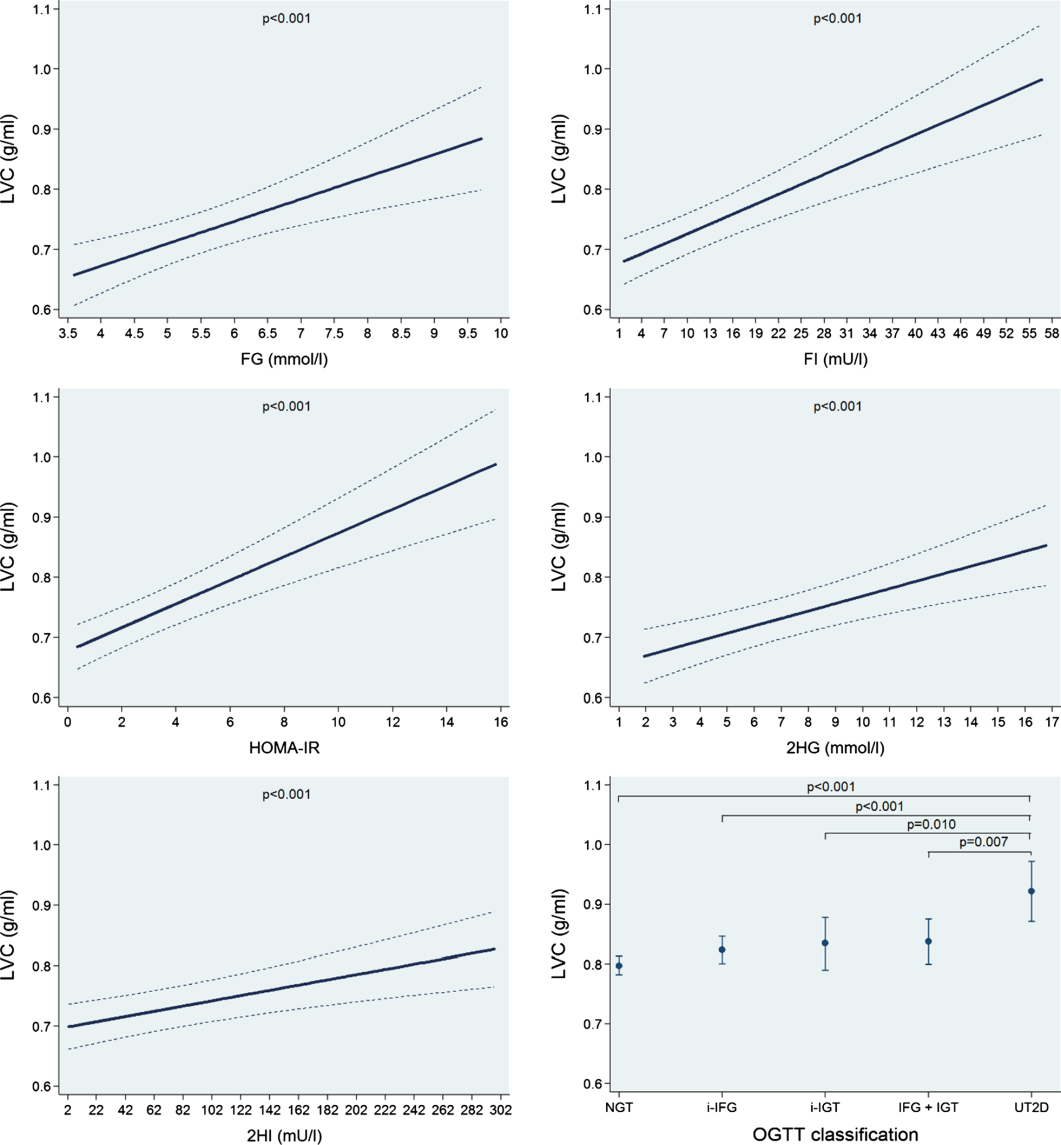
Adjusted* line (95% CI) showing the associations between fasting glucose (FG) and insulin (FI), the homeostasis model assessment-insulin resistance index (HOMA-IR) and 2-h postload glucose (2HG) and insulin (2HI) with left ventricular concentricity (LVC). Adjusted* mean (95% CI) LVC according to oral glucose tolerance test (OGTT) classification: normal glucose tolerance (NGT), isolated impaired fasting glucose (i-IFG), isolated impaired glucose tolerance (i-IGT), combined IFG and IGT (IFG + IGT) and unknown type 2 diabetes (UT2D). *Linear regression adjusted for age, sex, body fat-free mass, body fat mass, systolic blood pressure, use of antihypertensive medication, smoking status, alcohol consumption, sedentarism, estimated glomerular filtration rate, fasting time and study sample

In multivariable-adjusted regression analyses, we found no significant associations of FG, FI, HOMA-IR, 2HG and 2HI and the OGTT groups with LVMI (Additional file 1: Figure S1 and Table 2). We observed inverse linear associations of FG and 2HG with both LVEDVI and LVESVI, while FI, HOMA-IR and 2HI were inversely associated with these outcomes in a log-linear fashion. A 1 mmol/l higher FG was associated with a 1.18 ml/m^2.7^ (95% confidence interval: 0.57 to 1.80; p < 0.001) smaller LVEDVI and a 0.48 ml/m^2.7^ (0.12 to 0.84; p = 0.008) smaller LVESVI. We observed that the NGT group had larger LVEDVI and LVESVI mean values than the other groups, while in the group with UT2D adjusted mean values for LVEDVI and LVESVI were the smallest volumes (Fig. 1 and Additional file 1: Figure S2 and Table 2). In addition, we found positive linear associations of FG, FI, HOMA-IR, 2HG and 2HI with LVWTI. A 1 mmol/l higher FG was associated with a 0.042 mm/m^2.7^ (0.014 to 0.070; p = 0.003) higher LVWTI. The NGT group had the lowest LVWTI mean value and the UT2D group the largest one (Fig. 2 and Table 2). Finally, we also observed significant positive linear associations of FG, FI, HOMA-IR, 2HG and 2HI with LVC. A 1 mmol/l higher FG was associated with a 0.037 g/ml (0.018 to 0.056; p < 0.001) higher LVC. The UT2D group had the greatest mean adjusted LVC value (Fig. 3 and Table 2).

**Table 2:** Adjusted* β-coefficient (95% confidence interval [CI]) of the associations between fasting glucose (FG) and insulin (FI), the homeostasis model assessment-insulin resistance index (HOMA-IR) and 2-h postload glucose (2HG) and insulin (2HI) with left ventricular mass index (LVMI), left ventricular end-diastolic volume index (LVEDVI), left ventricular end-systolic volume index (LVESVI), left ventricular wall-thickness index (LVWTI) and left ventricular concentricity (LVC)

Parameter	Fasting glucose β-coefficient (95% CI), p-value (n = 1001)	Fasting insulin β-coefficient (95% CI), p-value (n = 1001)	HOMA-IR β-coefficient (95% CI), p-value (n = 999)	2-h glucose β-coefficient (95% CI), p-value (n = 1001)	2-h insulin β-coefficient (95% CI), p-value (n = 984)
Left ventricular mass index (g/m^2.7^)	− 0.08 (− 0.58 to 0.42), p = 0.761	0.01 (− 0.04 to 0.06), p = 0.761	0.05 (− 0.13 to 0.22), p = 0.602	− 0.03 (− 0.18 to 0.13), p = 0.726	− 0.00 (− 0.01 to 0.00), p = 0.324
Left ventricular end-diastolic volume index (ml/m^2.7^)	− 1.18 (− 1.80 to − 0.57), *p < 0.001*	− 2.42^a^ (− 3.17 to − 1.67), *p < 0.001*	− 2.27^a^ (− 2.96 to − 1.58), *p < 0.001*	− 0.35 (− 0.54 to − 1.16), *p < 0.001*	− 1.70^a^ (− 2.24 to − 1.17), *p < 0.001*
Left ventricular end-systolic volume index (ml/m^2.7^)	− 0.48 (− 0.84 to − 0.12), *p = 0.008*	− 1.19^a^ (− 1.65 to − 0.72), *p < 0.001*	− 1.10^a^ (− 1.53 to − 0.68), *p < 0.001*	− 0.18 (− 0.29 to − 0.07), *p = 0.002*	− 0.96^a^ (− 1.27 to − 0.66), *p < 0.001*
Left ventricular wall-thickness index (mm/m^2.7^)	0.042 (0.014 to 0.070), *p = 0.003*	0.007 (0.004 to 0.010), *p < 0.001*	0.025 (0.015 to 0.034), *p < 0.001*	0.013 (0.004 to 0.021), *p = 0.004*	0.001 (0.000 to 0.001), *p = 0.003*
Left ventricular concentricity	0.037 (0.018 to 0.056), *p < 0.001*	0.006 (0.004 to 0.007), *p < 0.001*	0.020 (0.013 to 0.026), *p < 0.001*	0.012 (0.007 to 0.018), *p < 0.001*	0.000 (0.000 to 0.001), *p < 0.001*

Italic values indicate significance of p-value < 0.05

* Linear regression adjusted for age, sex, body fat-free mass, body fat mass, systolic blood pressure, use of antihypertensive medication, smoking status, alcohol consumption, sedentarism, estimated glomerular filtration rate, fasting time and study sample

^a^Log-linear association

### Associations of FG, FI, HOMA-IR, 2HG and 2HI and the OGTT groups with arterial stiffness and LV systolic function

**Fig. 4:**
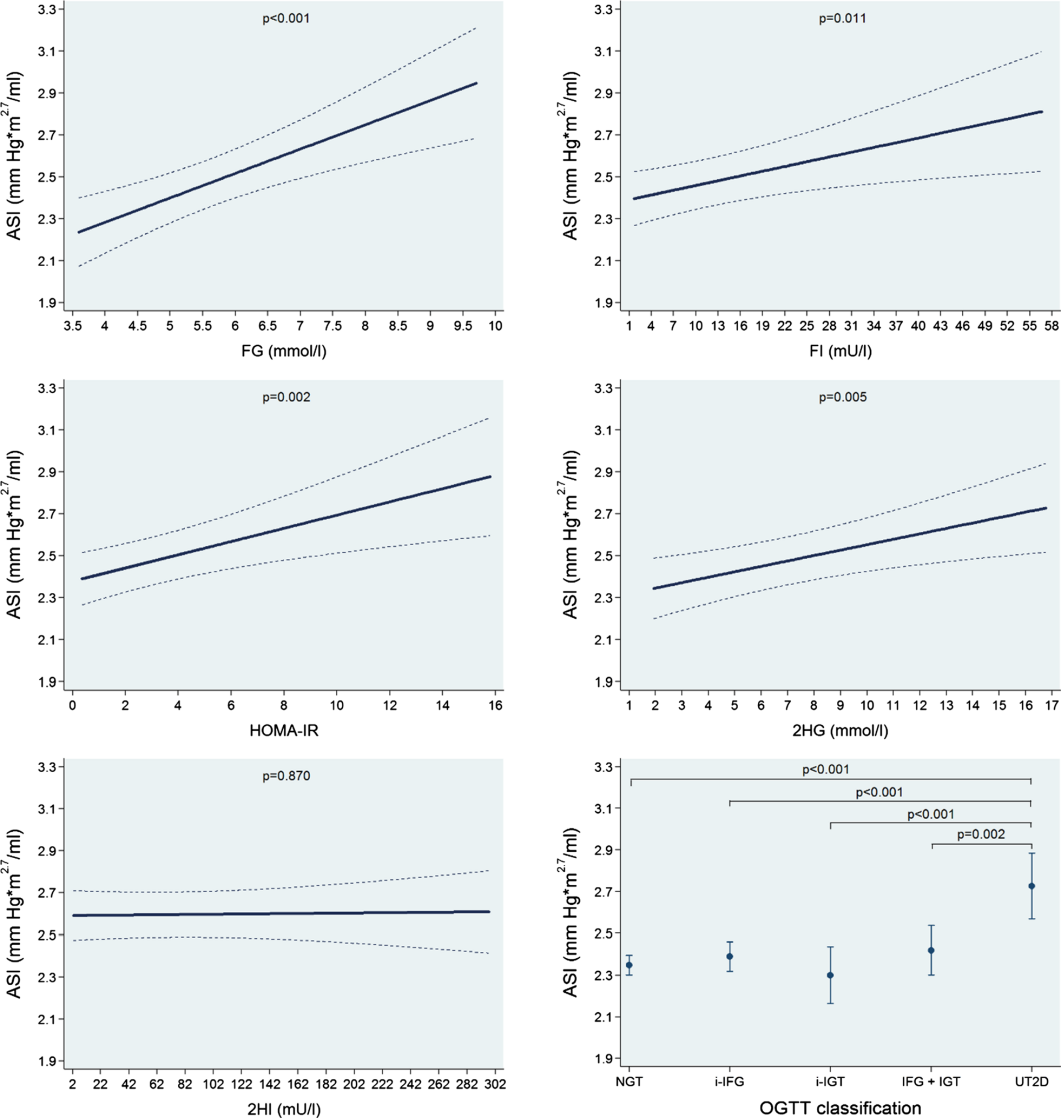
Adjusted* line (95% CI) showing the associations between fasting glucose (FG) and insulin (FI), the homeostasis model assessment-insulin resistance index (HOMA-IR) and 2-h postload glucose (2HG) and insulin (2HI) with arterial stiffness index (ASI). Adjusted* mean (95% CI) ASI according to oral glucose tolerance test (OGTT) classification: normal glucose tolerance (NGT), isolated impaired fasting glucose (i-IFG), isolated impaired glucose tolerance (i-IGT), combined IFG and IGT (IFG + IGT) and unknown type 2 diabetes (UT2D). *Linear regression adjusted for age, sex, body fat-free mass, body fat mass, systolic blood pressure, use of antihypertensive medication, smoking status, alcohol consumption, sedentarism, estimated glomerular filtration rate, fasting time and study sample

**Fig. 5:**
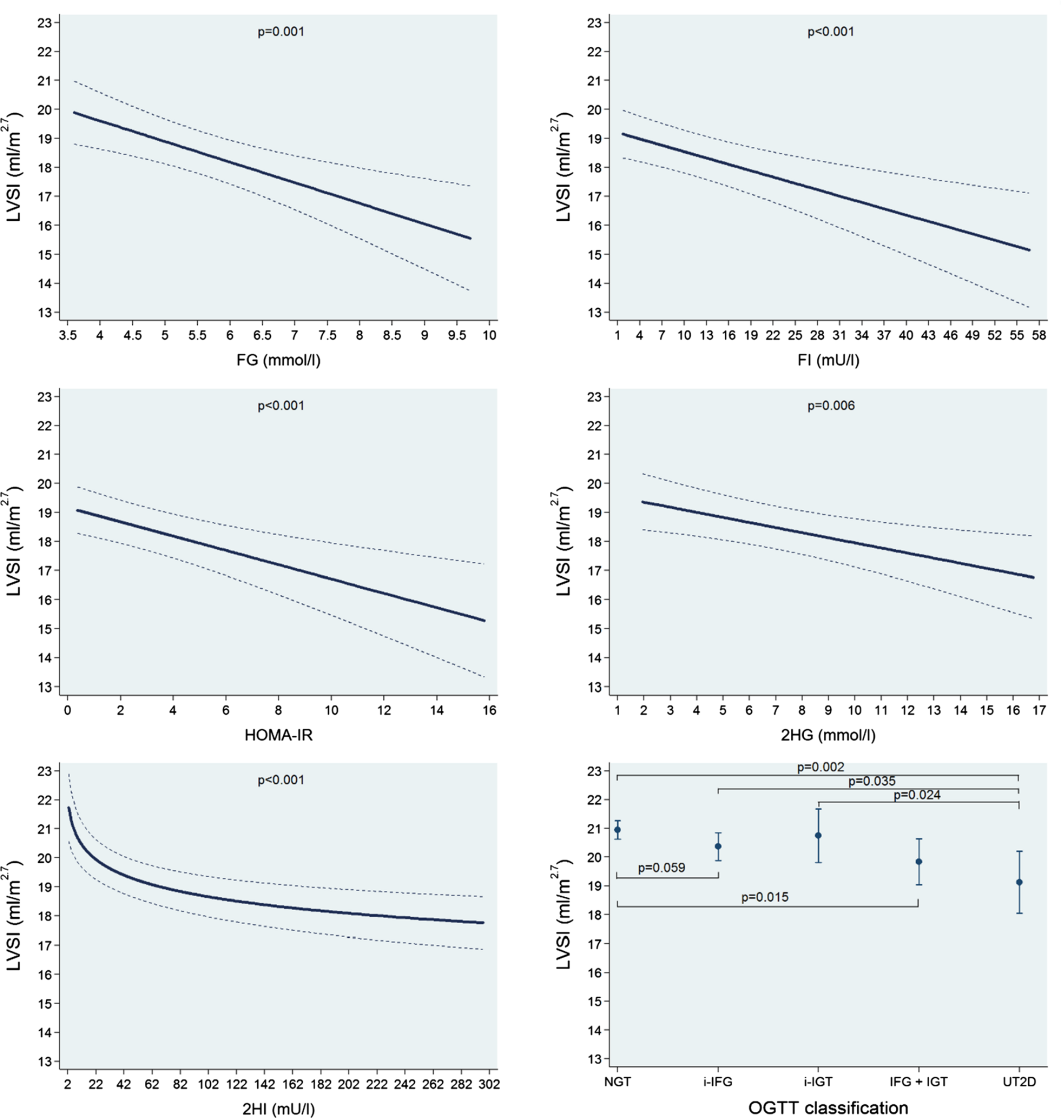
Adjusted* line (95% CI) showing the associations between fasting glucose (FG) and insulin (FI), the homeostasis model assessment-insulin resistance index (HOMA-IR) and 2-h postload glucose (2HG) and insulin (2HI) with left ventricular stroke volume index (LVSI). Adjusted* mean (95% CI) LVSI according to oral glucose tolerance test (OGTT) classification: normal glucose tolerance (NGT), isolated impaired fasting glucose (i-IFG), isolated impaired glucose tolerance (i-IGT), combined IFG and IGT (IFG + IGT) and unknown type 2 diabetes (UT2D). *Linear regression adjusted for age, sex, body fat-free mass, body fat mass, systolic blood pressure, use of antihypertensive medication, smoking status, alcohol consumption, sedentarism, estimated glomerular filtration rate, fasting time and study sample

In multivariable-adjusted regression analyses, we found significant positive linear associations of FG, FI, HOMA-IR and 2HG with ASI while no such association was observed for 2HI. A 1 mmol/l higher FG was associated with a 0.12 mmHg m^2.7^/ml (0.06 to 0.17; p < 0.001) higher ASI. Moreover, the UT2D group had the greatest mean ASI value (Fig. 4 and Table 3). On the other hand, we observed significant inverse linear associations of FG, FI, HOMA-IR and 2HG with LVSI, while 2HI was inversely associated with this outcome in a log-linear fashion. A 1 mmol/l higher FG was associated with a 0.71 ml/m^2.7^ (0.31 to 1.11; p = 0.001) lower LVSI. We also found that while the NGT group had the largest LVSI mean value than the other groups, the UT2D had the smallest one (Fig. 5 and Table 3). Alternatively, we saw positive linear associations of FG, FI, HOMA-IR, 2HG and 2HI with HR. The NGT group had the lowest HR mean value and the UT2D group the highest one (Additional file 1: Figure S3 and Table 3). Finally, we did not find significant associations of FG, FI, HOMA-IR, 2HG and 2HI and the OGTT groups with LVCI and just an isolated positive linear association of 2HI with LVEF. All the other associations with LVEF were not significant (Additional file 1: Figures S4, S5 and Table 3).

**Table 3:** Adjusted* β-coefficient (95% confidence interval [CI]) of the associations between fasting glucose (FG) and insulin (FI), the homeostasis model assessment-insulin resistance index (HOMA-IR) and 2-h postload glucose (2HG) and insulin (2HI) with arterial stiffness index (ASI), left ventricular stroke volume index (LVSI), heart rate (HR), left ventricular cardiac output index (LVCI) and left ventricular ejection fraction (LVEF)

Parameter	Fasting glucose β-coefficient (95% CI), p-value (n = 1001)	Fasting insulin β-coefficient (95% CI), p-value (n = 1001)	HOMA-IR β-coefficient (95% CI), p-value (n = 999)	2-h glucose β-coefficient (95% CI), p-value (n = 1001)	2-h insulin β-coefficient (95% CI), p-value (n = 984)
Arterial stiffness index (mmHg*m^2.7^/ml)	0.12 (0.06 to 0.17), *p < 0.001*	0.01 (0.00 to 0.01), *p = 0.011*	0.03 (0.01 to 0.05), *p = 0.002*	0.03 (0.01 to 0.04), *p = 0.005*	0.00 (− 0.00 to − 0.00), *p = 0.870*
Left ventricular stroke volume index (ml/m^2.7^)	− 0.71 (− 1.11 to − 0.31), *p = 0.001*	− 0.07 (− 0.11 to − 0.03), *p < 0.001*	− 0.25 (− 0.38 to − 0.11), *p < 0.001*	− 0.18 (− 0.30 to − 0.05), *p = 0.006*	− 0.84^a^ (− 1.17 to − 0.50), *p < 0.001*
Heart rate (bpm)	2.13 (0.91 to 3.34), *p = 0.001*	4.92^a^ (3.34 to 6.51), *p < 0.001*	4.49^a^ (3.06 to 5.93), *p < 0.001*	0.68 (0.34 to 1.05), *p < 0.001*	0.03 (0.02 to 0.05), *p < 0.001*
Left ventricular cardiac output index (l/min*m^2.7^)	− 0.02 (− 0.05 to − 0.01), p = 0.305	0.00 (− 0.00 to 0.00), p = 0.690	0.00 (− 0.01 to 0.01), p = 0.875	− 0.00 (− 0.01 to 0.01), p = 0.681	− 0.00 (− 0.00 to 0.00), p = 0.423
Left ventricular ejection fraction (%)	0.01 (− 0.66 to 0.67), p = 0.982	0.06 (− 0.00 to 1.13), p = 0.061	0.20 (− 0.03 to 0.43), p = 0.088	0.10 (− 0.11 to 0.30), p = 0.350	0.01 (0.00 to 0.02), *p = 0.003*

Italic values indicate significance of p-value < 0.05

* Linear regression adjusted for age, sex, body fat-free mass, body fat mass, systolic blood pressure, use of antihypertensive medication, smoking status, alcohol consumption, sedentarism, estimated glomerular filtration rate, fasting time and study sample

^a^Log-linear association

## Discussion

In our analyses we found inverse associations of FG, FI, HOMA-IR, 2HG and 2HI with LVEDVI in individuals without known diabetes. FG mainly represents nocturnal hepatic gluconeogenesis dependent on hepatic insulin sensitivity and 2HG mainly reflects postprandial hyperglycemia. Conversely, we found positive linear associations of these glycemic variables with LVWTI. The net result of the associations of higher values of glycemic indicators on the left ventricular geometry was a greater left ventricular concentricity. This concentric remodeling was independent of other determinants, such as hypertension.

Noteworthy, the higher values of the glycemic variables were also accompanied by greater values of ASI and lower values of LVSI. The higher values of HR observed in association with the greater values of the glycemic variables might be a compensatory mechanism, for the lower LVSI, trying to avoid any deleterious consequence on the LVCI which was, in reality, not affected as well as the LVEF. This means that subjects with higher values of the glycemic variables would have a smaller “reserve”, in this circumstance heart rate, to utilize under stress when compared with individuals with lower levels.

In summary, our findings showed a direct relation between higher glucose and/or insulin levels and greater arterial stiffness, smaller LV chamber size and higher LV thickness with resultant LV concentric remodeling and lower LV stroke volume. These changes in heart geometry and function may be related to the development of heart failure with preserved ejection fraction (HFpEF).

### In the context of the published literature

A previous analysis of the Multiethnic Study of Atherosclerosis (MESA) [4] showed that individuals with IFG had smaller LVEDV and LVSV and no significant difference regarding LVM, LVCO and LVEF, when compared with normoglycemic subjects. When diabetic subjects were compared with persons with normoglycemia, besides the smaller LVEDV and LVSV, the LVEF was lower and the LVM was higher. A more recent analysis of the MESA study [5] demonstrated that IFG and HOMA-IR were positively associated with LVC. Moreover, in univariate analyses stratified by BMI, subjects with IFG had a higher LVC and LVMI and a lower LVEDVI when compared with individuals with normal fasting glucose.

An investigation of 1603 individuals with a mean age of 64 years from the Framingham Heart Study [6], demonstrated positive associations in age-adjusted models of HOMA-IR with LVMI, LVC, relative wall thickness, LVCO and LVEF for men and women. Noteworthy, after further adjustments, that included BMI, just LVC remained positively associated with HOMA-IR, while the relation with LVMI became even an inverse one.

A previous investigation of the Atherosclerosis Risk in Communities (ARIC) study [7] showed that prediabetes and type 2 diabetes were associated with higher arterial stiffness when compared with subjects with normal glucose levels. A recent study [32] suggests that insulin resistance might be an early marker of arterial stiffness in healthy and active young to middle-age men. Triglyceride glucose index is the product of fasting plasma glucose and triglycerides and is a strong surrogate for insulin resistance [33]. Previous studies found that the triglyceride glucose index was positive associated with the risk for incident type 2 diabetes [34] and with arterial stiffness in a relatively healthy Korean population [35] and in lean postmenopausal women [33]. Another study [36] indicated that lipopolysaccharide-binding protein levels, a surrogate of inflammation immune responses, were associated with arterial stiffness among male patients with type 2 diabetes independently of obesity and traditional cardiovascular risk factors. Interestingly, treatment with liraglutide in patients with recently diagnosed type 2 diabetes reduced oxidative stress resulting in an improvement of arterial stiffness and left ventricular myocardial strain [37]. Moreover, in an experimental model with induced type 2 diabetes in female mice, empagliflozin improved kidney injury by promoting glycosuria, and probably by reducing systemic and renal artery stiffness [38].

Finally, our group has previously published findings from two independent analyses of KORA FF4 samples. The first one [15] which used multivariate models without adjustment for height, weight or BMI, showed that individuals with prediabetes and type 2 diabetes had lower LVEDV, LVESV, LVSV and higher LVM and LVEF (just individuals with prediabetes) when compared with subjects with normal glucose metabolism. The second one [16], which further adjusted for BMI, showed that individuals with prediabetes and type 2 diabetes had higher LVWT when compared with subjects with normal glucose metabolism. We did not find any other MRI study regarding LVWT.

All the previously cited studies included MRI determined heart parameters. The results of our current analyses are, in general, in line with these studies regarding the findings of associations of higher glycemic and insulin levels with lower LV cavity size and greater wall thickness, concentricity and arterial stiffness without effect on LVCO and LVEF. On the other hand, compared with previous studies, we did not find any association of the OGTT parameters with LVMI. We believe that the main reason for this specific finding was the use of body fat-free and fat mass, instead of BMI, as covariate (after the initial normalization of cardiac parameters to height^2.7^). As far as we know, almost all previous studies that analyzed the associations of glucose and/or insulin levels with cardiac structure and function by echocardiography or MRI utilized BMI as a covariate. BIA assess body composition which is considered a better measurement of obesity than BMI as it allows to differentiate between fat-free and fat mass which both contribute to BMI [39]. In sensitivity analyses (Additional file 1: Table S4), we compared three regression models of the associations of HOMA-IR with LVMI. In the first model, besides the adjustment for age, sex, systolic blood pressure, use of antihypertensive medication, smoking status, alcohol consumption, sedentarism, estimated glomerular filtration rate, fasting time and study sample we further adjusted for body fat-free and fat mass (our original model). In the second model, we did not adjust for fat mass and fat-free-mass, but for BMI. Finally, in the third model we further adjusted for weight and height. Remarkably, while in our original model we had no significant association (p = 0.602), it was highly significant, when adjusted for BMI and the association was an inverse one (Additional file 1: Table S4). This seems to be the same phenomenon that has been observed in the above-discussed analysis of the Framingham Heart Study [6]. Actually, we believe that adjustment for BMI (which includes height in its calculation) in a model that included a variable already normalized to height^2.7^, might represent an over adjustment and thus, potentially, might lead to a misleading result. The reason for our choice of body fat-free and fat mass, instead of BMI, as cofounders in the multivariate models was the potential diverse effects of the different components of the body composition. Body fat-free and fat mass might have diverse effects on the OGTT parameters and, at the same time, the LV structure and function explainable by their different structural composition, metabolic demands and functional manifestations. Body fat-free mass is responsible for almost all of the body’s metabolic requirements while body fat mass may be accountable for the release of numerous biomarkers and inflammatory cytokines. Moreover, body fat-free mass might have a metabolic protective effect that mitigates the excess of the previously mentioned markers. We believe that our approach to normalize the cardiac parameters to height^2.7^ and subsequent adjust for body fat-free and fat mass was the most feasible strategy to analyze the effects of OGTT parameters on the heart independently of obesity which is highly correlated with both exposures and outcomes.

### Potential mechanisms for the observed associations

Glycemic disorders and insulin resistance are usually accompanied by several cardiovascular and metabolic risk factors and co-morbidities like older age, obesity, hypercholesterolemia, hypertension and coronary heart disease. Otherwise, the involved pathologic mechanisms that might explain these associations are still not completely clarified [2]. Our study protocol was not designed to elucidate possible pathophysiological mechanisms that might be involved in the associations described in our analyses and the cross-sectional design of our study restricts the evaluation of causal relationships. Besides that, there is still no prospective clinical trial that had undoubtedly demonstrated that higher glucose and insulin levels might have a causal association with changes in the heart [2]. Nevertheless, we have integrated various risk factors in our multivariable regression models. Our results might support a direct relation between higher glucose and/or insulin levels and greater arterial stiffness, smaller LV chamber size and higher LV thickness with resultant LV concentric remodeling and lower LV stroke volume. The LV cardiac output would be kept due to higher heart rate.

Dose–response modeling results suggest that fasting glycemic levels do not have a clearly defined threshold in their relations with cardiac parameters, as with retinopathy, but rather a continuous association. Hyperglycemia and hyperinsulinemia are usually accompanied by increased free fatty acid levels, systemic and tissue inflammation, oxidative stress, and activation of the renin–angiotensin–aldosterone system and the sympathetic nervous system. Otherwise, the effects of increased glycemic and insulinemic levels on the cardiac structure are, in the beginning, clinically asymptomatic [2]. These initial effects are characterized by higher stiffness (mainly by increased intracellular Ca^2+^), increased collagen and advanced glycation end products, fibrosis and cellular hypertrophy (mainly by expression of hypertrophic genes) [2]. Noteworthy, this process should be considered as not specific of the heart, but rather of the entire cardio-vascular system. Because of this initial process, the LV chamber size decreases and the LV wall hypertrophies leading to cardiac remodeling, cardiac diastolic dysfunction and eventually systolic dysfunction. Our analyses are in line with the subclinical presentation of this process.

Finally, one of the possible complications that may affect individuals with type 2 diabetes and even with prediabetes is a cardiovascular autonomic neuropathy. This condition is characterized by sinus tachycardia, exercise intolerance, and orthostatic hypotension [40]. Moreover, it may also be associated with left ventricular systolic and diastolic dysfunction independent of any other cardiac disease [41]. Interestingly, while the cardiovascular autonomic neuropathy might result in a decrease in the left ventricular filing volume, leading to a lower stroke volume, it also causes sinus tachycardia that will result in a normal cardiac output. Unfortunately, our study protocol did not include measurements, such as heart rate variation, the Valsalva maneuver and postural changes in blood pressure to evaluate in more detail the cardiac autonomic function.

### Study limitations

We need to mention some limitations of our analyses. First, our study sample consisted of European Caucasians; therefore, further analyses of samples with different ethnicity and age groups would be desirable to investigate the strength of this association across those groups. Second, the cross-sectional design is a limitation, which means that relationships between cause and effect might be not recognized. Third, our sample consisted of data from two separate studies (SHIP-TREND-0 and KORA FF4) with some minor methodological variances including difference in sample ages (21 to 81 in SHIP-TREND-0 and 39 to 73 in KORA FF4). While we consider that this did not have an effect on our results, we cannot conclusively exclude it (even after supplementary adjustment for study sample in our regression models). Finally, although we have incorporated numerous confounders into our multivariable regression models, we cannot exclude residual confounding due to unmeasured conditions.

However, our analyses also have some noteworthy strengths, including the large number of subjects based on two cohorts of the general population, the standardized evaluation of OGTT data after an overnight fast, and the availability of lifestyle data and multiple metabolic risk factors.

## Conclusions

Our results showed inverse associations of FG (consequence of nocturnal hepatic gluconeogenesis), FI, HOMA-IR, 2HG (result of postprandial hyperglycemia) and 2HI with LV chamber size. On the other hand, these glycemic variables were positively associated with LV wall thickness resulting in a LV concentric remodeling pattern. Moreover, higher values of the glycemic variables were also accompanied by greater values of arterial stiffness and lower values of LV stroke volume, but not with changes in LV cardiac output (since an accompanying higher heart rate) and LV ejection fraction.

## Supplementary information

**Additional file 1.** Expanded methods, additional figures and tables.

## Data Availability

The datasets generated during and/or analyzed during the current study are not publicly available due to data protection aspects but are available in an anonymized form from the corresponding author on reasonable request.

## Abbreviations

2HG: 2-h postload glucose (2HG) and insulin (2HI)
2HI: 2-h postload insulin
ADA: American Diabetes Association
ARIC: Atherosclerosis Risk in Communities
ASI: arterial stiffness index
BIA: bioelectrical impedance analysis
BMI: lower body mass index
DM: diabetes mellitus
eGFR: estimated glomerular filtration rate
FG: fasting glucose
FI: fasting insulin
FFM: body fat-free mass
FM: body fat mass
HOMA-IR: homeostasis model assessment-insulin resistance index
IFG: impaired fasting glucose
IGT: impaired glucose tolerance
i-IFG: isolated impaired fasting glucose
i-IGT: isolated impaired glucose
KORA: Kooperative Gesundheitsforschung in der Region Augsburg-Cooperative Health Research in the Region of Augsburg
KORA FF4: second follow-up of the population-based KORA S4 cohort
LV: left ventricular
LVC: left ventricular concentricity
LVCO: left ventricular cardiac output
LVCI: left ventricular cardiac output index
LVEDV: left ventricular end-diastolic
LVEDVI: left ventricular end-diastolic index
LVEF: left ventricular ejection fraction
LVESV: left ventricular end-systolic volume
LVESVI: left ventricular end-systolic volume index
LVM: left ventricular mass
LVMI: left ventricular mass index
LVSV: left ventricular stroke volume
LVSI: left ventricular stroke volume index
LVWT: left ventricular wall-thickness
LVWTI: left ventricular wall-thickness index
MESA: Multiethnic Study of Atherosclerosis
MRI: magnetic resonance imaging
NGT: normal glucose tolerance
NHANES: National Health and Nutrition Examination Survey
OGTT: oral glucose tolerance test
SHIP: Study of Health in Pomerania
SHIP-TREND-0: baseline examination of the Study of Health in Pomerania TREND-0 cohort
SSFP: steady-state free precession
UT2D: unknown type 2 diabetes
