# Supplementary material for: Glucose and insulin levels are associated with arterial stiffness and concentric remodeling of the heart
Source: Cardiovasc Diabetol. 2019 Nov 4;18:145. doi: 10.1186/s12933-019-0948-4 (PMC6829934; doi:10.1186/s12933-019-0948-4)
Supplement: Supplementary file 1 — Additional file 1. Expanded methods, additional figures and tables. [file 12933_2019_948_MOESM1_ESM.docx]

**Additional material**

**Expanded Methods**

**Study population**

The present cross-sectional study is based on data from two population-based investigations, the Study of Health in Pomerania (SHIP) and the Cooperative Health Research in the Region of Augsburg (KORA - Kooperative Gesundheitsforschung in der Region Augsburg).

**SHIP-TREND-0 (Northeastern Germany)**

The sample of the study is a subsample derived from data from a second independent cohort, called SHIP-TREND-0 [[1](#_ENREF_1)] which was established to cover the same region as the initial SHIP sample. The study design and recruitment strategy have been described in more detail elsewhere [[1](#_ENREF_1), [2](#_ENREF_2)]. In brief, a random cluster sample (age range 20 to 79 years) was taken from the population of West Pomerania, the northeastern region of Germany. The net sample (without migrated or deceased persons) included 8,826 adult individuals with 4,420 (2,275 women, 51.5%) of them participating in the baseline (SHIP-TREND-0) study (response 50.1%) conducted between 2008 and 2012. Of these, 991 subjects (435 women, 43.9%), aged 21 to 81 years, who were eligible and willing to undergo whole-body MRI participated in the MRI substudy.

**KORA FF4 (Southern Germany)**

The study sample KORA FF4 is a subsample of the second follow-up of the population-based KORA S4 cohort, a population-based health survey conducted in the city of Augsburg and 16 municipalities from the surrounding counties (about 600,000 inhabitants), a southern region of Germany. Recruitment and eligibility criteria for the KORA studies have been described elsewhere [[3](#_ENREF_3)]. In brief, a two-stage cluster sampling method was used which followed the WHO MONICA project method in the Augsburg region. For Augsburg city, a simple random sampling was performed. In the surrounding rural districts, 16 counties were selected with probabilities proportional to their size. A total sample of 6,640 subjects was drawn from the target population consisting of German residents of the region aged 25 to 74 years. Out of these, 4,261 participated in the baseline examination (KORA S4) between 1999 and 2001 (response 64.2%) [[4](#_ENREF_4)]. From them 3,080 subjects were included in the KORA F4 examination that was conducted between 2006 and 2008. The KORA FF4 study was conducted between 2013 and 2014 and included 2,279 of the originally recruited 4,261 KORA S4 participants. Of these, 400 subjects (169 women, 42.3%), aged 39 to 73 years, who were eligible and willing to undergo whole-body MRI participated in the MRI substudy. The detailed participant flow and exclusion criteria have been described previously [[5](#_ENREF_5)].

**Pooled sample**

Our pooled sample, from SHIP-TREND-0 and KORA FF4, comprised 1,391 individuals (604 women, 43.4%) aged 21 to 81 years. Individuals with inadequate image quality (n=79), previous myocardial infarction or stroke (n=16), left ventricular ejection fraction (determined by MRI) less than 40% (n=9), fasting time less than 8 hours (n=217), use of hypoglycemic medication (n=37), missing values for OGTT parameters (n=17) or any of the covariates (n=9) as well as individuals with extreme values (>99.5th percentile for fasting glucose, insulin or 2-hour postload glucose; n=6) were excluded. Accordingly, our final analytical sample consisted of 1,001 individuals (453 women, 45.3%), aged 21 to 80 years.

All study participants gave written informed consent. The study was approved by the ethics committees of the University of Greifswald, the Bavarian Chamber of Physicians, and the Ludwig-Maximilians-Universität München and complies with the Declaration of Helsinki.

**Glucose and insulin measurements, oral glucose tolerance test and classification of prediabetes and unknown type 2 diabetes**

In both studies, blood was collected without stasis from the antecubital vein by a trained examiner following a standardized protocol, refrigerated to 4–8 °C and shipped on refrigerant packaging within 4 to a maximum of 6 h to the laboratory.[[6](#_ENREF_6)] Measurements of fasting glucose (FG) and 2-hour postload glucose (2HG) were based on plasma in SHIP-TREND-0 and on serum in KORA FF4. In order to determine whether plasma and serum samples were comparable, duplicate measurements were carried out using serum samples from all SHIP-TREND-0 participants. Both measurements (plasma and serum glucose) were highly correlated (r=0.99; p<0.0001). In Passing Bablok regression analysis of serum versus plasma glucose (mmol/l) an intercept of -0.10 (95%CI: -0.10 to -0.10) and a slope of 1 were observed concluding that plasma values in SHIP-TREND-0 might be only slightly lower than serum values. Furthermore, a previous analysis[[6](#_ENREF_6)] with 30 serum blood glucose samples were drawn at random from the first follow-up of the population-based KORA S4 cohort, KORA F4, and reassessed in the SHIP-TREND-0 laboratory, yielding a correlation coefficient of r=0.94. On average, these original 30 KORA F4 measurements were only slightly lower (mean -0.06 mmol/l; SD 0.17) when they were re-analyzed in the SHIP-TREND-0 laboratory. Thus, serum glucose from KORA FF4 and plasma glucose from SHIP-TREND-0 were considered as comparable for the current analysis.

In both studies, FG was sampled and 75 grams of anhydrous glucose (Dextro OGT; Boehringer Mannheim, Mannheim, Germany) was given to those participants without diagnosed type 2 diabetes or taking glucose-lowering agents. In SHIP-TREND-0, plasma FG and 2HG levels were measured using a hexokinase method (Dimension Vista 1500, Siemens Healthcare Diagnostics, Eschborn, Germany) [[6](#_ENREF_6)]  and serum fasting insulin (FI) and 2-hour postload glucose insulin (2HI) values were measured by an electrochemiluminescence immunoassay (ADVIA Centaur, Siemens Healthcare Diagnostics, Eschborn, Germany) [[7](#_ENREF_7)]. In KORA FF4, serum FG and 2HG levels were measured using an enzymatic colorimetric method (Dimension Vista 1500, Siemens Healthcare Diagnostics, Eschborn, Germany or Cobas c702, Roche Diagnostics GmbH, Mannheim, Germany) and FI and 2HI values were measured by a solid-phase enzyme-labeled chemiluminescent immunometric assay (Immulite 2000 Xpi, Siemens Healthcare Diagnostics, Eschborn, Germany) or by an electrochemiluminescence immunoassay (Cobas e 602, Roche Diagnostics GmbH, Mannheim, Germany). Halfway during the study, measurement instruments and assays changed over from Siemens to Roche. Siemens measurements for insulin (in µU/ml) were calibrated to Roche measurements based on 194 samples which were measured with both instruments using the following formula: Insulin_Roche = 1.307 + (Insulin_Siemens * 1.016). Glucose measurements were not recalibrated because the two measurement methods showed high agreement as determined by Pasisng.Bablok regression [[8](#_ENREF_8)].

The homeostasis model assessment-insulin resistance index (HOMA-IR) was calculated as (FG [mmol/l] X FI [μU/ml]) / 22.5 [[9](#_ENREF_9)].

Following the criteria of the American Diabetes Association (ADA) [[10](#_ENREF_10)], we classified individuals as having normal glucose tolerance (NGT) when they had FG values <5.6 mmol/l (<100 mg/dl) and 2HG <7.8 mmol/l (<140mg/dl). Unknown type 2 diabetes (UT2D) was defined as FG values ≥7.0 mmol/l (≥126 mg/dl) or 2HG ≥11.1 mmol/l (≥200mg/dl). Participants were clssified as having prediabetes if FG values were between 5.6 and 6.9 mmol/l (100-125 mg/dl, impaired fasting glucose: IFG) and/or 2HG values were between 7.8 and 11.0 mmol/l (140-199 mg/dl, impaired glucose tolerance: IGT) [[6](#_ENREF_6), [10](#_ENREF_10)]. We defined three groups of prediabetes: isolated impaired fasting glucose (i-IFG), isolated impaired glucose tolerance (i-IGT), and combined IFG and IGT (IFG + IGT) [[6](#_ENREF_6), [10](#_ENREF_10)].

**Cardiac MR Imaging**

In SHIP-TREND, cardiac MR imaging was performed on a 1.5 Tesla MR system (Magnetom Avanto; Siemens Medical Systems, Erlangen, Germany) [[7](#_ENREF_7), [11](#_ENREF_11)] and in KORA FF4, on a 3 Tesla MR system (Magnetom Skyra; Siemens Medical Systems, Erlangen, Germany) [[5](#_ENREF_5), [8](#_ENREF_8)]. In both studies imaging of cardiac function and morphology was performed using cine steady-state free precession (cine-SSFP) sequences. In SHIP-TREND-0 with a voxel size of 2.0 x 1.4 x 6.0 mm and 1.0 mm slice gap. SSFP cine long-axes (4- and 2-chamber view) were acquired with a repetition time (TR) of 2.65 ms; echo time (TE) of 1.12 ms; 66° flip angle; field of view 340 x 276; matrix, 192 x 125; bandwith, 930 Hz/Pixel. SSFP cine short-axes were acquired using a TR of 2.82 ms; TE of 1.20 ms; 68° flip angle; field of view, 360 x 292; matrix, 256 x 146; bandwith, 977 Hz/Pixel. Transverse SSFP cine views were acquired with a TR of 56.2 ms; TE of 1.18 ms; 68° flip angle; field of view, 292 x 360; matrix, 256 x 146; bandwith, 930 Hz/Pixel. Postcontrast images were acquired 15 minutes after intravenous injection of 0.15 ml/kg body weight of gadobutrol (Gadovist, Bayer Schering Healthcare, Leverkusen, Germany) as phase-sensitive inversion recovery single-shot SSFP sequence in short-axis orientation (TR of 2.4 ms; TE of 1.03 ms; 40° flip angle; field of view, 299 x 399; matrix, 192 x 101; voxel size of 3.0 x 2.1 x 6.0 mm; inversion time 280-320 msec; bandwith, 1184 Hz/Pixel). In KORA FF4 with a stack of 10 layer and 25 phases per cardiac cycle as well as in a 4-chamber view (echo time 1.46 ms, repetition time 29.97 ms, in-plane voxel size 1.5 × 1.5 mm, flip angle 62–63°, field-of-view 297 × 360 mm, matrix size 240 × 160 mm, slice thickness 8 mm). Late Gadolinium enhancement was determined on Fast Low Angle Shot (FLASH) inversion recovery sequences 10 minutes after administration of 0.15 ml/kg of body weight of gadopentetate dimeglumine (Gadovist, Bayer Healthcare, Leverkusen, Germany) with the parameters TR of 700-1000 ms; TE of 1.55 ms; 20-55° flip angle; field of view: 300 x 360; matrix: 256 x 140; voxel size of 3.0 x 2.1 x 6.0 mm)

**Image Analysis**

In SHIP-TREND-0, quantitative image analysis was performed by two observers, unaware of the subject’s glycemic status and all other clinical covariates, with 3 and 5 years of cardiac MR imaging experience using semiautomatic tools in QMass MR 7.2 (MEDIS, Leiden, Netherlands). Interobserver variability was computed in a random subsample of 5%. Post-contrast images were interpreted by the two readers mentioned above and supervised in a consensus reading by a radiologist with 12 years of experience. All observers were unaware of the participants’ medical history [[11](#_ENREF_11)]. In KORA FF4, the cine-SSFP sequences were analysed semi-automatically using cvi42 software (Circle Cardio-vascular Imaging, Calgary, Canada) by two readers unaware of the subject’s glycemic status and all other clinical covariates. Intraobserver agreement was assessed on 25 randomly chosen subjects that were evaluated twice by the first reader. To assess Interobserver agreement, 52 subjects were evaluated the first and the second reader. Intra- and Interobserver agreement were calculated by the Intraclass Correlation Coefficient (ICC).

For LV analysis, in both SHIP-TREND-0 and KORA FF4, contours of end-diastolic and end-systolic endocardial as well as epicardial borders were marked in long-axis and a stack of short-axis images. Based on the biplane area–length method from apical 4-and 2-chamber views, the software generated a volume/time curve during the cardiac cycle. LV end-diastolic volume (LVEDV) was determined during the first image of the acquisition. LV end-systolic volume (LVESV) was measured by determining the phase in which the LV intra-cavity blood pool was at its smallest by visual assessment at the midventricular level. LV myocardial mass (LVM) was calculated at the end-diastole using the specific density of the myocardium (1.05 g/cm^3^) [[11](#_ENREF_11)]. Papillary muscles were included in the LVM and excluded of the LV end-diastolic and systolic volumes. Basal slices were included if at least half of the LV circumference blood pool was confined by myocardium [[12](#_ENREF_12)]. Inclusion or exclusion of apical slices depended on the visibility of myocardium. LV wall-thickness (LVWT) was determined in the 16-segment model (according to the AHA-segment model).[[13](#_ENREF_13)] LV concentricity (LVC) was calculated as LVM / LVEDV. LV stroke volume (LVSV), LV cardiac output (LVCO) and LV ejection fraction (LVEF) were calculated following the formulas below:

LVSV (ml) = LVEDV – LVESV

LVCO (l/min) = LVSV x heart rate

LVEF (%) = (LVEDV - LVESV) / LVEDV

LVM, LVEDV, LVESV, LVWT, LVSV and LVCO were indexed for body height in meters, normalized to the allometric power of 2.7, which linearizes the relations between the cardiac anatomic and functional parameters with height and identifies the impact of obesity [[14](#_ENREF_14)]. This resulted in LVM index (LVMI), LVEDV index (LVEDVI), LVESV index (LVESVI), LVWT index (LVWTI), LVSV index (LVSI) and LVCO index (LVCI).

Arterial stiffness index (ASI) was calculated as (systolic blood pressure – diastolic blood pressure) / LVSI [[15](#_ENREF_15)].

**Interview, medical and laboratory examinations**

In both studies, information on socio-economic variables (including years of school education [<10, 10, or >10 years]), smoking status (never, former or current smoker) [[16](#_ENREF_16)], alcohol consumption (in grams per day) and medical history was collected by trained and certificated medical staff during a standardized interview. Sedentary lifestyle was defined as individuals who did not participate in leisure time exercise, for at least one hour per week, during summer or winter [[17](#_ENREF_17)]. Participants were asked to bring the original packaging of their medications that were taken during the last 7 days before the examination date. Unique identifiers and drug names were recorded according to the ATC classification system.

All participants underwent an extensive standardized medical examination. Anthropometric measurements included height and weight based on recommendations of the World Health Organization (WHO) [[18](#_ENREF_18)]. Weight was measured to the nearest 0.1 kg in light clothing and without shoes using standard digital scales. Body mass index (BMI) was calculated as weight (kg)/height² (m²). Waist circumference (WC) was measured to the nearest 0.1 cm using an inelastic tape midway between the lower rib margin and the iliac crest in the horizontal plane, with the participant standing comfortably with weight distributed evenly on both feet [[19](#_ENREF_19)]. While in SHIP-TREND-0 body fat–free mass (FFM) and fat mass (FM) were measured by bioelectrical impedance analysis (BIA) using a multifrequency Nutriguard-M device (Data Input, Pöcking, Germany) and the NUTRI4 software (Data Input, Pöcking, Germany) [[20-22](#_ENREF_20)], in KORA FF4, BIA scans were obtained by BIA 2000-S device (Data Input, Pöcking, Germany) with an operating frequency of 50 kHz at 0.8 mA. Ohmic resistance was measured at the dominant hand (between wrist and dorsum) and the dominant foot (between angle and dorsum).

After a resting period of at least five minutes, systolic and diastolic blood pressures as well as heart rate were measured three times on the right arm of seated subjects using an oscillometric digital blood pressure monitor (HEM-705CP, Omron Corporation, Tokyo, Japan) with an interval of three minutes between readings. The mean of the second and third measurements was used for the present analyses. Antihypertensive medication was defined as use of agents with the ATC-code C02, C03, C07, C08 and C09 [[23](#_ENREF_23)]. Hypertension was defined as systolic blood pressure ≥140 mm Hg and/or diastolic blood pressure ≥90 mm Hg and/or current self-reported use of any anti-hypertensive medications.

Fasting blood samples were obtained from all study participants while sitting [[24](#_ENREF_24)]. In SHIP-TREND-0, glycated hemoglobin was determined by high-performance liquid chromatography (Diamat, Bio-Rad Laboratories, Munich, Germany). Total serum cholesterol, low-density lipoprotein cholesterol (LDL-C) and high-density lipoprotein cholesterol (HDL-C) were measured photometrically (Dimension RxL or Dimension VISTA 1500, Siemens Healthcare Diagnostics, Eschborn, Germany). Serum creatinine concentration was assessed using a modified kinetic Jaffé method (Dimension RxL or Dimension Vista 1500, Siemens Healthcare Diagnostics, Eschborn, Germany). In KORA FF4, glycated hemoglobin was measured in hemolyzed whole blood using the cation-exchange high performance liquid chromatographic, photometric VARIANT II TURBO HbA1c Kit - 2.0 assay on a VARIANT II TURBO Hemoglobin Testing System (Bio-Rad Laboratories Inc., Hercules, USA). Total serum cholesterol, low-density lipoprotein cholesterol (LDL-C), high-density lipoprotein cholesterol (HDL-C) and serum creatinine concentrations were measured using an enzymatic colorimetric method (Dimension Vista 1500, Siemens Healthcare Diagnostics, Eschborn, Germany or Cobas c702, Roche Diagnostics GmbH, Mannheim, Germany). Because of the changes from Siemens to Roche, the Siemens measurement results were calibrated to the Roche measurements using the following formulas (in mg/dL): Total_Cholesterol_Roche = 3.00 + (Total_Cholesterol_Siemens * 1.00); HDL_Cholesterol_Roche = 2.40 + (HDL_Cholesterol_Siemens * 1.12); LDL_Cholesterol_Roche = antilog (- 0.13328 + [log LDL_Cholesterol_Siemens * 1.03051]); Creatine_Roche = - 0.037568 + (Creatinine_Siemens * 1.02703) [[8](#_ENREF_8)].

Hypercholesterolemia was defined as use of lipid-lowering medication defined by the ATC-code C10 and/or total serum cholesterol ≥ 6.2 mmol/l and/or LDL-C ≥ 4.1 mmol/l and/or total cholesterol/HDL-C ratio ≥ 5.0. The estimated glomerular filtration rate was estimated according to the CKD-EPI formula [[25](#_ENREF_25)] and expressed in ml/min/1.73 m².

**Statistical analysis**

To characterize the study population, data was reported as median (with 25^th^ and 75^th^ percentiles) for continuous variables and as percentages for categorical variables stratified by OGTT classification.

We used linear regression models to associate FG, FI, HOMA-IR, 2HG and 2HI levels and OGTT groups with LVMI, LVEDVI, LVESVI, LVWTI, LVC, ASI, LVSI, HR, LVCI and LVEF. The basic multivariable models were adjusted for age, sex, body fat-free mass, body fat mass, systolic blood pressure, use of antihypertensive medication, smoking status, alcohol consumption, sedentarism, estimated glomerular filtration rate and fasting time. Because of differences in crude values regarding clinical characteristics and parameters of left ventricular geometry and function between the two studies (**Additional Table S1, S2 and S3**), the pooled analyses were additionally adjusted for study sample (SHIP-TREND-0, KORA FF4). We used fractional polynomials to test potential non-linear relationships between exposure and outcomes [[26](#_ENREF_26)].

A two-sided p-value p<0.05 was considered as statistically significant. Statistical analyses were performed using Stata 14.2 (Stata Corporation, College Station, TX, USA).

**Figure S1:**

Adjusted* line (95% CI) showing the associations between fasting glucose (FG) and insulin (FI), the homeostasis model assessment-insulin resistance index (HOMA-IR) and 2-hour postload glucose (2HG) and insulin (2HI) with left ventricular mass index (LVMI). Adjusted* mean (95% CI) LVMI according to oral glucose tolerance test (OGTT) classification: normal glucose tolerance (NGT), isolated impaired fasting glucose (i-IFG), isolated impaired glucose tolerance (i-IGT), combined IFG and IGT (IFG + IGT) and unknown type 2 diabetes (UT2D).


*Linear regression adjusted for age, sex, body fat-free mass, body fat mass, systolic blood pressure, use of antihypertensive medication, smoking status, alcohol consumption, sedentarism, estimated glomerular filtration rate, fasting time and study sample.

**Figure S2:**

Adjusted* line (95% CI) showing the associations between fasting glucose (FG) and insulin (FI), the homeostasis model assessment-insulin resistance index (HOMA-IR) and 2-hour postload glucose (2HG) and insulin (2HI) with left ventricular end-systolic volume index (LVESVI). Adjusted* mean (95% CI) LVESVI according to oral glucose tolerance test (OGTT) classification: normal glucose tolerance (NGT), isolated impaired fasting glucose (i-IFG), isolated impaired glucose tolerance (i-IGT), combined IFG and IGT (IFG + IGT) and unknown type 2 diabetes (UT2D).

*****Linear regression adjusted for age, sex, body fat-free mass, body fat mass, systolic blood pressure, use of antihypertensive medication, smoking status, alcohol consumption, sedentarism, estimated glomerular filtration rate, fasting time and study sample.

**Figure S3:**

Adjusted* line (95% CI) showing the associations between fasting glucose (FG) and insulin (FI), the homeostasis model assessment-insulin resistance index (HOMA-IR) and 2-hour postload glucose (2HG) and insulin (2HI) with heart rate (HR). Adjusted* mean (95% CI) HR according to oral glucose tolerance test (OGTT) classification: normal glucose tolerance (NGT), isolated impaired fasting glucose (i-IFG), isolated impaired glucose tolerance (i-IGT), combined IFG and IGT (IFG + IGT) and unknown type 2 diabetes (UT2D).

*****Linear regression adjusted for age, sex, body fat-free mass, body fat mass, systolic blood pressure, use of antihypertensive medication, smoking status, alcohol consumption, sedentarism, estimated glomerular filtration rate, fasting time and study sample.**Figure S4:**

Adjusted* line (95% CI) showing the associations between fasting glucose (FG) and insulin (FI), the homeostasis model assessment-insulin resistance index (HOMA-IR) and 2-hour postload glucose (2HG) and insulin (2HI) with left ventricular cardiac output index (LVCI). Adjusted* mean (95% CI) LVCI according to oral glucose tolerance test (OGTT) classification: normal glucose tolerance (NGT), isolated impaired fasting glucose (i-IFG), isolated impaired glucose tolerance (i-IGT), combined IFG and IGT (IFG + IGT) and unknown type 2 diabetes (UT2D).

*Linear regression adjusted for age, sex, body fat-free mass, body fat mass, systolic blood pressure, use of antihypertensive medication, smoking status, alcohol consumption, sedentarism, estimated glomerular filtration rate, fasting time and study sample.

**Figure S5:**

Adjusted* line (95% CI) showing the associations between fasting glucose (FG) and insulin (FI), the homeostasis model assessment-insulin resistance index (HOMA-IR) and 2-hour postload glucose (2HG) and insulin (2HI) with left ventricular ejection fraction (LVEF). Adjusted* mean (95% CI) LVEF according to oral glucose tolerance test (OGTT) classification: normal glucose tolerance (NGT), isolated impaired fasting glucose (i-IFG), isolated impaired glucose tolerance (i-IGT), combined IFG and IGT (IFG + IGT) and unknown type 2 diabetes (UT2D).


*Linear regression adjusted for age, sex, body fat-free mass, body fat mass, systolic blood pressure, use of antihypertensive medication, smoking status, alcohol consumption, sedentarism, estimated glomerular filtration rate, fasting time and study sample.

**Table S1:** Characteristics of the study sample stratified by study sample.

| **Parameter** | **SHIP-TREND-0** | **KORA FF4** | **p-value*** |
| --- | --- | --- | --- |
| **N (%)** | 656 (65.5%) | 345 (34.5%) |  |
| **Age (years)** | 49 (39,59) | 55 (47, 63) | **<0.001** |
| **Women (%)** | 46.3 | 43.2 | 0.341 |
| **Fasting serum glucose (mmol/l)** | 5.4 (5.0, 5,7) | 5.4 (5.1, 5.9) | 0.093 |
| **2-hours postload serum glucose (mmol/l)** | 6.0 (5.1, 7,3) | 5.8 (4.7, 7.3) | 0.105 |
| **Fasting insulin (µlU/ml)** | 8.5 (5.9, 13.1) | 9.2 (6.3, 13.5) | 0.180 |
| **2-hours postload insulin (µlU/ml)** | 48.1 (30.4, 86.0) | 46.0 (26.7, 79.0) | 0.071 |
| **Homeostasis model assessment-insulin resistance index (HOMA-IR)** | 2.03 (1.39, 3.25) | 2.20 (1.44, 3.52) | 0.109 |
| **Glycated hemoglobin (%)** | 5.2 (4.9, 5.5) | 5.4 (5.3, 5.6) | **<0.001** |
| **Estimated glomerular filtration rate (ml/min/1.73 m²)** | 93.9 (82.7, 104) | 87.7 (78.7, 96.0) | **<0.001** |
| **Smoking (%)** |  |  |  |
| Never | 42.5 | 36.8 |  |
| Current | 20.0 | 20.9 |  |
| Former | 37.5 | 42.3 | 0.197 |
| **Alcohol consumption (g/day)** | 4.12 (1.42, 11.4) | 8.79 (1.29, 26.6) | **0.003** |
| **Weight (kg)** | 78.9 (69.2, 89.9) | 81.5 (71.6, 92.8) | 0.051 |
| **Height (cm)** | 171.(165, 179) | 172 (164, 179) | 0.535 |
| **Body mass index (kg/m^2^)** | 26.9 (24.2, 29.8) | 27.2 (24.4, 30.8) | 0.095 |
| **Body fat-free mass (kg)** | 57.2 (47.3, 66.9) | 56.0 (46.1, 64.4) | **0.026** |
| **Body fat mass (kg)** | 21.0 (16.8, 27.1) | 24.8 (20.5, 30.9) | **<0.001** |
| **Waist circumference (cm)** | 88.8 (79.5, 97.3) | 98.6 (88.5, 107) | **<0.001** |
| **Systolic blood pressure (mm Hg)** | 126 (114, 137) | 120 (108, 130) | **<0.001** |
| **Diastolic blood pressure (mm Hg)** | 76.5 (70.5, 83.0) | 75.0 (68.5, 80.5) | **0.001** |
| **Hypertension (%)** | 39.6 | 31.6 | **0.012** |
| **Antihypertensive medications (%)** | 24.4 | 23.5 | 0.748 |
| **Total cholesterol (mmol/l)** | 5.40 (4.80, 6.20) | 5.63 (5.01, 6.25) | **0.013** |
| **Hypercholesterolemia (%)** | 40.2 | 42.9 | 0.417 |
| **Lipid-lowering medication (%)** | 6.40 | 8.12 | 0.312 |
| **Sedentarism (%)** | 29.1 | 37.7 | **0.006** |

Data are medians (25th, 75th percentile) or percentage.

*p-values are based on the chi-squared test for categorical variables and the Wilcoxon rank-sum (or Mann-Whitney) tests for continuous variables.

**Table S2:** Crude values of left ventricular mass index (LVMI), left ventricular end-diastolic volume index (LVEDVI), left ventricular end-systolic volume index (LVESVI), left ventricular wall-thickness index (LVWTI) and left ventricular concentricity (LVC) stratified by study sample.

| **Parameter** | **SHIP-TREND-0** | **KORA FF4** | **p-value*** |
| --- | --- | --- | --- |
| **N (%)** | 656 (65.5%) | 345 (34.5%) |  |
| **Left ventricular mass index (g/m^2.7^)** | 22.8 (19.5, 25.9) | 31.5 (28.0, 35.7) | **<0.001** |
| **Left ventricular end-diastolic volume index (ml/m^2.7^)** | 35.0 (31.2, 38.9) | 30.2 (25.9, 34.2) | **<0.001** |
| **Left ventricular end-systolic volume index (ml/m^2.7^)** | 14.5 (12.3, 16.6) | 8.86 (6.86, 11.3) | **<0.001** |
| **Left ventricular wall-thickness index (mm/m^2.7^)** | 1.90 (1.68, 2.17) | 2.74 (2.42, 3.12) | **<0.001** |
| **Left ventricular concentricity** | 0.65 (0.58, 0.73) | 1.04 (0.89, 1.26) | **<0.001** |

Data are medians (25th, 75th percentile).

*p-values are based on the Wilcoxon rank-sum (or Mann-Whitney) tests.

**Table S3:** Crude values of left arterial stiffness index (ASI), left ventricular stroke volume index (LVSI), heart rate (HR), left ventricular cardiac output index (LVCI) and left ventricular ejection fraction (LVEF) stratified by study sample.

| **Parameter** | **SHIP-TREND-0** | **KORA FF4** | **p-value*** |
| --- | --- | --- | --- |
| **N (%)** | 656 (65.5%) | 345 (34.5%) |  |
| **Arterial stiffness index (mm Hg*m^2.7^/ml)** | 2.36 (1.94, 2.83) | 2.10 (1.70, 2.59) | **<0.001** |
| **Left ventricular stroke index (ml/m^2.7^)** | 20.4 (17.8, 22.8) | 20.7 (17.6, 23.5) | 0.261 |
| **Heart rate (bpm)** | 76 (68, 83) | 66 (60, 73) | **<0.001** |
| **Left ventricular cardiac index (l/min*m^2.7^)** | 1.52 (1.35, 1.71) | 1.36 (1.16, 1.52) | **<0.001** |
| **Left ventricular ejection fraction (%)** | 58.4 (54.5, 62.4) | 70.2 (64.5, 74.6) | **<0.001** |

Data are medians (25th, 75th percentile).

*p-values are based on the Wilcoxon rank-sum (or Mann-Whitney) tests.

**Table S4:** Adjusted* ß-coefficient (95% confidence interval [CI]) of the associations between the homeostasis model assessment-insulin resistance index (HOMA-IR) with left ventricular mass index (LVMI) with further supplementary adjustments for body fat-free and fat mass, body mass index and weight and height.

| **Parameter** | **HOMA-IR**  **ß-coefficient (95% CI), p-value**  **(adjusted for body fat-free and fat mass)** | **HOMA-IR**  **ß-coefficient (95% CI), p-value**  **(adjusted for body mass index)** | **HOMA-IR**  **ß-coefficient (95% CI), p-value**  **(adjusted for weight and height)** |
| --- | --- | --- | --- |
| **Left ventricular mass index (g/m^2.7^)** | 0.05 (-0.13 to 0.22), p=0.602 | -0.23 (-0.39 to -0.07), **p=0.005** | -0.20 (-0.36 to -0.04), **p=0.014** |

*Linear regression adjusted for age, sex, systolic blood pressure, use of antihypertensive medication, smoking status, alcohol consumption, sedentarism, estimated glomerular filtration rate, fasting time and study sample.

**References**

1. Volzke H, Alte D, Schmidt CO, Radke D, Lorbeer R, Friedrich N, Aumann N, Lau K, Piontek M, Born G *et al*: **Cohort profile: the study of health in Pomerania**. *Int J Epidemiol* 2011, **40**(2):294-307.

2. John U, Greiner B, Hensel E, Ludemann J, Piek M, Sauer S, Adam C, Born G, Alte D, Greiser E *et al*: **Study of Health In Pomerania (SHIP): a health examination survey in an east German region: objectives and design**. *Sozial- und Praventivmedizin* 2001, **46**(3):186-194.

3. Holle R, Happich M, Lowel H, Wichmann HE, Group MKS: **KORA--a research platform for population based health research**. *Gesundheitswesen* 2005, **67 Suppl 1**:S19-25.

4. Rathmann W, Haastert B, Icks A, Lowel H, Meisinger C, Holle R, Giani G: **High prevalence of undiagnosed diabetes mellitus in Southern Germany: target populations for efficient screening. The KORA survey 2000**. *Diabetologia* 2003, **46**(2):182-189.

5. Bamberg F, Hetterich H, Rospleszcz S, Lorbeer R, Auweter SD, Schlett CL, Schafnitzel A, Bayerl C, Schindler A, Saam T *et al*: **Subclinical Disease Burden as Assessed by Whole-Body MRI in Subjects With Prediabetes, Subjects With Diabetes, and Normal Control Subjects From the General Population: The KORA-MRI Study**. *Diabetes* 2017, **66**(1):158-169.

6. Tamayo T, Schipf S, Meisinger C, Schunk M, Maier W, Herder C, Roden M, Nauck M, Peters A, Volzke H *et al*: **Regional differences of undiagnosed type 2 diabetes and prediabetes prevalence are not explained by known risk factors**. *PLoS One* 2014, **9**(11):e113154.

7. Markus MRP, Ittermann T, Wittfeld K, Schipf S, Siewert-Markus U, Bahls M, Bulow R, Werner N, Janowitz D, Baumeister SE *et al*: **Prediabetes is associated with lower brain gray matter volume in the general population. The Study of Health in Pomerania (SHIP)**. *Nutrition, metabolism, and cardiovascular diseases : NMCD* 2017, **27**(12):1114-1122.

8. Rospleszcz S, Schafnitzel A, Koenig W, Lorbeer R, Auweter S, Huth C, Rathmann W, Heier M, Linkohr B, Meisinger C *et al*: **Association of glycemic status and segmental left ventricular wall thickness in subjects without prior cardiovascular disease: a cross-sectional study**. *BMC Cardiovasc Disord* 2018, **18**(1):162.

9. Huth C, Beuerle S, Zierer A, Heier M, Herder C, Kaiser T, Koenig W, Kronenberg F, Oexle K, Rathmann W *et al*: **Biomarkers of iron metabolism are independently associated with impaired glucose metabolism and type 2 diabetes: the KORA F4 study**. *European journal of endocrinology* 2015, **173**(5):643-653.

10. American Diabetes A: **2. Classification and Diagnosis of Diabetes**. *Diabetes Care* 2017, **40**(Suppl 1):S11-S24.

11. Bulow R, Ittermann T, Dorr M, Poesch A, Langner S, Volzke H, Hosten N, Dewey M: **Reference ranges of left ventricular structure and function assessed by contrast-enhanced cardiac MR and changes related to ageing and hypertension in a population-based study**. *Eur Radiol* 2018, **28**(9):3996-4005.

12. Juergens KU, Grude M, Maintz D, Fallenberg EM, Wichter T, Heindel W, Fischbach R: **Multi-detector row CT of left ventricular function with dedicated analysis software versus MR imaging: initial experience**. *Radiology* 2004, **230**(2):403-410.

13. Hogan JW, Roy J, Korkontzelou C: **Handling drop-out in longitudinal studies**. *Statistics in medicine* 2004, **23**(9):1455-1497.

14. de Simone G, Devereux RB, Kimball TR, Mureddu GF, Roman MJ, Contaldo F, Daniels SR: **Interaction between body size and cardiac workload: influence on left ventricular mass during body growth and adulthood**. *Hypertension* 1998, **31**(5):1077-1082.

15. Devereux RB, Roman MJ, Paranicas M, O'Grady MJ, Lee ET, Welty TK, Fabsitz RR, Robbins D, Rhoades ER, Howard BV: **Impact of diabetes on cardiac structure and function: the strong heart study**. *Circulation* 2000, **101**(19):2271-2276.

16. Maziak W, Hense HW, Doring A, Keil U: **Ten-year trends in smoking behaviour among adults in southern Germany**. *The international journal of tuberculosis and lung disease : the official journal of the International Union against Tuberculosis and Lung Disease* 2002, **6**(9):824-830.

17. Markus MR, Lieb W, Stritzke J, Siewert U, Troitzsch P, Koch M, Dorr M, Felix SB, Volzke H, Schunkert H *et al*: **Light to Moderate Alcohol Consumption Is Associated With Lower Risk of Aortic Valve Sclerosis: The Study of Health in Pomerania (SHIP)**. *Arteriosclerosis, thrombosis, and vascular biology* 2015, **35**(5):1265-1270.

18. **Physical status: the use and interpretation of anthropometry. Report of a WHO Expert Committee**. *World Health Organization technical report series* 1995, **854**:1-452.

19. Baumeister SE, Friedrich N, Schmidt CO, Volzke H, Nauck M, Hoffmann W, Flessa S, Marschall P, Wallaschofski H: **Association of IGF-I and IGFBP-3 with health care costs and hospitalization: results from a prospective observational study**. *Growth Horm IGF Res* 2011, **21**(2):89-95.

20. Kyle UG, Bosaeus I, De Lorenzo AD, Deurenberg P, Elia M, Gomez JM, Heitmann BL, Kent-Smith L, Melchior JC, Pirlich M *et al*: **Bioelectrical impedance analysis--part I: review of principles and methods**. *Clin Nutr* 2004, **23**(5):1226-1243.

21. Kusztal M, Kleszczynski J, Weyde W, Makulska I, Porazko T, Golebiowski T, Krajewska M, Zwolinska D, Klinger M: **Pulse volume changes recorded by air plethysmography during single hemodialysis sessions**. *Blood purification* 2008, **26**(6):498-504.

22. Kohler A, King R, Bahls M, Gross S, Steveling A, Gartner S, Schipf S, Glaser S, Volzke H, Felix SB *et al*: **Cardiopulmonary fitness is strongly associated with body cell mass and fat-free mass: The Study of Health in Pomerania (SHIP)**. *Scandinavian journal of medicine & science in sports* 2018, **28**(6):1628-1635.

23. WHO/ISH-Guidelines-Subcommittee: **1999 World Health Organization-International Society of Hypertension Guidelines for the Management of Hypertension. Guidelines Subcommittee**. *Journal of hypertension* 1999, **17**(2):151-183.

24. Baumeister SE, Volzke H, Marschall P, John U, Schmidt CO, Flessa S, Alte D: **Impact of fatty liver disease on health care utilization and costs in a general population: a 5-year observation**. *Gastroenterology* 2008, **134**(1):85-94.

25. Levey AS, Stevens LA, Schmid CH, Zhang YL, Castro AF, 3rd, Feldman HI, Kusek JW, Eggers P, Van Lente F, Greene T *et al*: **A new equation to estimate glomerular filtration rate**. *Annals of internal medicine* 2009, **150**(9):604-612.

26. Sauerbrei W, Meier-Hirmer C, Benner A, Royston P: **Multivariable regression model building by using fractional polynomials: Description of SAS, STATA and R programs**. *Comput Stat Data An* 2006, **50**(12):3464-3485.
